# *Candida albicans* Inhibits *Pseudomonas aeruginosa* Virulence through Suppression of Pyochelin and Pyoverdine Biosynthesis

**DOI:** 10.1371/journal.ppat.1005129

**Published:** 2015-08-27

**Authors:** Eduardo Lopez-Medina, Di Fan, Laura A. Coughlin, Evi X. Ho, Iain L. Lamont, Cornelia Reimmann, Lora V. Hooper, Andrew Y. Koh

**Affiliations:** 1 Department of Pediatrics, University of Texas Southwestern Medical Center, Dallas, Texas, United States of America; 2 Department of Biochemistry, University of Otago, Dunedin, New Zealand; 3 Department of Fundamental Microbiology, University of Lausanne, Lausanne, Switzerland; 4 Department of Immunology, University of Texas Southwestern Medical Center, Dallas, Texas, United States of America; 5 The Howard Hughes Medical Institute, University of Texas Southwestern Medical Center, Dallas, Texas, United States of America; 6 Center for Genetics of Host Defense, University of Texas Southwestern Medical Center, Dallas, Texas, United States of America; 7 Department of Microbiology, University of Texas Southwestern Medical Center, Dallas, Texas, United States of America; 8 Harold C. Simmons Comprehensive Cancer Center, University of Texas Southwestern Medical Center, Dallas, Texas, United States of America; Geisel School of Medicine at Dartmouth, UNITED STATES

## Abstract

Bacterial-fungal interactions have important physiologic and medical ramifications, but the mechanisms of these interactions are poorly understood. The gut is host to trillions of microorganisms, and bacterial-fungal interactions are likely to be important. Using a neutropenic mouse model of microbial gastrointestinal colonization and dissemination, we show that the fungus *Candida albicans* inhibits the virulence of the bacterium *Pseudomonas aeruginosa* by inhibiting *P*. *aeruginosa* pyochelin and pyoverdine gene expression, which plays a critical role in iron acquisition and virulence. Accordingly, deletion of both *P*. *aeruginosa* pyochelin and pyoverdine genes attenuates *P*. *aeruginosa* virulence. Heat-killed *C*. *albicans* has no effect on *P*. *aeruginosa*, whereas *C*. *albicans* secreted proteins directly suppress *P*. *aeruginosa* pyoverdine and pyochelin expression and inhibit *P*. *aeruginosa* virulence in mice. Interestingly, suppression or deletion of pyochelin and pyoverdine genes has no effect on *P*. *aeruginosa*’s ability to colonize the GI tract but does decrease *P*. *aeruginosa*’s cytotoxic effect on cultured colonocytes. Finally, oral iron supplementation restores *P*. *aeruginosa* virulence in *P*. *aeruginosa* and *C*. *albicans* colonized mice. Together, our findings provide insight into how a bacterial-fungal interaction can modulate bacterial virulence in the intestine. Previously described bacterial-fungal antagonistic interactions have focused on growth inhibition or colonization inhibition/modulation, yet here we describe a novel observation of fungal-inhibition of bacterial effectors critical for virulence but not important for colonization. These findings validate the use of a mammalian model system to explore the complexities of polymicrobial, polykingdom infections in order to identify new therapeutic targets for preventing microbial disease.

## Author Summary

*Pseudomonas aeruginosa* and *Candida albicans* are two medically important human pathogens that often co-infect or co-colonize the same human niches, such as the gut. In a normal healthy host, *P*. *aeruginosa* and *C*. *albicans* can colonize the gut without any significant pathologic sequelae. But in immunocompromised hosts, both pathogens can escape the gut and cause life-threatening disseminated infections. Yet the mechanisms and pathogenic consequences of interactions between these two pathogens within a living mammalian host are not well understood. Here, we use a mouse model of *P*. *aeruginosa* and *C*. *albicans* gut *co*-infection to better understand the mechanisms by which *C*. *albicans* inhibits *P*. *aeruginosa* infection. *C*. *albicans* inhibits the expression of *P*. *aeruginosa* genes that are vital for iron acquisition. Accordingly, deleting these iron acquisition genes in *P*. *aeruginosa* prevents infection. Understanding how microbes interact and antagonize each other may help us identify new potential therapeutic targets for preventing or treating infections.

## Introduction

The bacterium *Pseudomonas aeruginosa* and the fungus *Candida albicans*, two medically important human pathogens, co-infect or co-colonize numerous sites on the human body, including the gut [1], lung (including respiratory ventilator) [2–4], burn wound [5], genitourinary tract [1], and skin sites (including vascular catheters) [6,7]. *In vitro* studies suggest that mutually antagonistic interactions take place between *P*. *aeruginosa* and *C*. *albicans*. *P*. *aeruginosa* modulates *C*. *albicans* morphology [8] and can kill *C*. *albicans* filaments [9,10]. *C*. *albicans* inhibits *P*. *aeruginosa* cellular signaling [11] and metabolite production [11]. Although many clinical studies report the observation of mixed infections with *P*. *aeruginosa* and *C*. *albicans* [1,3,12,13], the impact on bacterial and/or fungal pathogenesis is still unclear [14,15].

In cancer and stem cell transplant patients, invasive *P*. *aeruginosa* and *C*. *albicans* infections are thought to arise from initial GI colonization and subsequent translocation after medically induced immune deficits [16–20]. Three primary defense mechanisms that prevent microbial translocation from the GI tract in humans and mice include 1) a stable gut microbiota; 2) intact intestinal mucosal barriers; and 3) intact host immune defenses, particularly cellular immunity [21–23]. Bacterial-fungal interactions can significantly impact gut microbiota homeostasis and gut mucosal integrity. For instance, bacteria can inhibit *C*. *albicans* GI colonization [24–26] and conversely, *C*. *albicans* modulates bacterial repopulation in the gut [27,28]. Importantly, the risk for bacteremia in cancer patients is directly proportional to gut bacterial burden [29]. Furthermore, bacteria and fungi can damage epithelial barriers by production of cytotoxic effector molecules (e.g. Type III secretion system in *P*. *aeruginosa*) [30–32] and morphology (yeast to hyphal transition in *C*. *albicans*) [33–36]. Thus bacterial-fungal interactions that affect these virulence determinants could significantly impact the pathogenesis of invasive disease.

Another line of host defense against microbial pathogen infection is the withholding of nutrients (such as iron) to prevent microbial overgrowth [37], a strategy known as nutritional immunity [38]. Since iron is not freely available in the mammalian host, most bacterial pathogens utilize high-affinity iron uptake mechanisms that compete against host-mediated sequestration of iron [38]. Accordingly, *P*. *aeruginosa* produces low-molecular weight secreted molecules known as siderophores (pyochelin and pyoverdine) that specifically chelate iron (Fe^3+^). Both pyochelin and pyoverdine have been shown to be important for virulence in pulmonary and burn wound models of *P*. *aeruginosa* infection [39–42]. *C*. *albicans* possesses similar iron acquisition mechanisms [43,44]. Thus, in iron-limited environments, such as the mammalian gut, the ability of one microbe (e.g. *C*. *albicans*) to prevent a competing microbe (e.g. *P*. *aeruginosa*) from acquiring iron could provide a significant fitness advantage.

To study the impact of fungi on bacterial virulence, we created a murine model of *P*. *aeruginosa* and *C*. *albicans* GI co-colonization and neutropenia-induced *P*. *aeruginosa* virulence. While *C*. *albicans* had no effect on *P*. *aeruginosa* GI colonization, *C*. *albicans* repressed expression of *P*. *aeruginosa* pyochelin and pyoverdine biosynthesis genes. Of note, the presence of *C*. *albicans* did not increase gut iron levels. Accordingly, deletion of both pyochelin and pyoverdine genes attenuated *P*. *aeruginosa* virulence. *C*. *albicans* secreted proteins were sufficient to inhibit *P*. *aeruginosa* pyochelin and pyoverdine gene expression and decrease *P*. *aeruginosa*’s cytotoxic effect on colonocytes *in vitro*. Strikingly, oral administration of *C*. *albicans* secreted proteins protected mice from *P*. *aeruginosa* infection. Finally, supplementation with oral iron restored *P*. *aeruginosa* virulence in *P*. *aeruginosa* and *C*. *albicans* colonized mice. Thus, by exploring bacterial-fungal interactions in the mammalian GI tract, we can identify new strategies for preventing invasive microbial infections.

## Results

### *C*. *albicans* inhibits *P*. *aeruginosa* virulence in neutropenic mice

**Fig 1:**
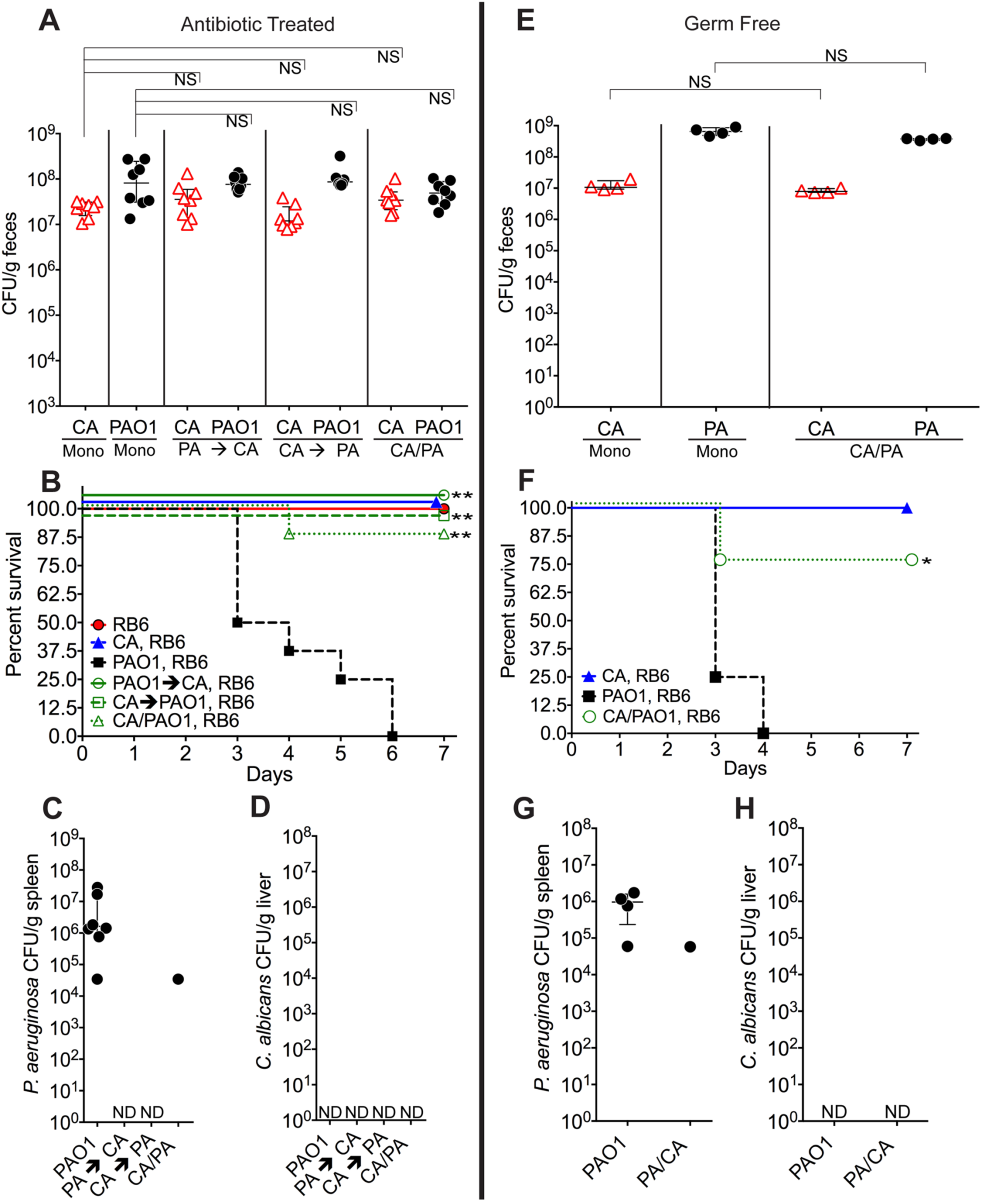
*C*. *albicans* inhibits *P*. *aeruginosa* virulence in mice. **A**, **E**) *C*. *albicans* SC5314 (red triangles) and *P*. *aeruginosa* PAO1 (black circles) GI colonization levels in (**A**) adult antibiotic-treated mice (C3H/HeN) and (**E**) germ-free adult mice (C57BL/6). n = 8 per group for antibiotic-treated mice. n = 4 per group for germ-free mice. Points represent results from individual animals. Horizontal lines with bars represent the median with interquartile range. Statistical analysis performed by Mann-Whitney test. **B**, **F**) Survival curves of neutropenic antibiotic-treated (**B**) and germ-free (**F**) mice colonized with *P*. *aeruginosa* ± *C*. *albicans*. Mice were made neutropenic with intraperitoneal injection of 0.200 mg RB6-8C5 rat anti-mouse Ly-6G, Ly-6C monoclonal antibody. n = 8 for antibiotic-treated mice. n = 4 for germ-free mice. Statistical analysis performed by log-rank test. **C, D, G, H**) *P*. *aeruginosa* and *C*. *albicans* levels in spleens (**C**, **G**) and livers (**D**, **H**) of deceased neutropenic antibiotic-treated and germ-free mice colonized with *P*. *aeruginosa* ± *C*. *albicans*. Organ homogenates were plated on cetrimide, MacConkey (aerobic), TSA (aerobic,), BHI/Blood (anaerobic), and YVG (YPD agar with vancomycin and gentamicin) plates. The presence of a homogeneous population of green, oxidase-positive colonies on cetrimide agar and an absence of other bacterial growth on the MacConkey, TSA and BHI/Blood plates was used for confirmation of *P*. *aeruginosa* dissemination. The presence of a homogeneous population of creamy white colonies on YVG agar was used for confirmation of *C*. *albicans* dissemination. Points represent results from individual animals. Horizontal lines with bars represent the median with interquartile range. * p< 0.05; ** p<0.01; ns, not significant. CA, *C*. *albicans*. PA, *P*. *aeruginosa*. RB6, RB6-8C5 monoclonal antibody.

We adapted a well-established murine model using oral antibiotic treatment to promote *C*. *albicans* [24,25] and *P*. *aeruginosa* colonization [31] and monoclonal antibody induced neutropenia to promote *P*. *aeruginosa* virulence only [31]. The presence of *C*. *albicans* SC5314 in the GI tract did not significantly affect *P*. *aeruginosa* PAO1 GI colonization levels compared to mice that had been mono-colonized with PAO1 (Fig 1A). The temporal sequence of GI colonization (*P*. *aeruginosa* first, *C*. *albicans* first, or *P*. *aeruginosa* and *C*. *albicans* simultaneously) did not affect *P*. *aeruginosa* GI colonization levels (Fig 1A). Conversely, the presence of *P*. *aeruginosa* did not significantly affect *C*. *albicans* colonization levels compared to mice mono-colonized with *C*. *albicans* (Fig 1A). In contrast, when *P*. *aeruginosa* and a single bacterial species (*Enterococcus faecalis*, *Bacteroides thetaiotaomicron*, *Escherichia coli*, or *Blautia producta*) were simultaneously introduced into the GI tract, *P*. *aeruginosa* levels significantly decreased compared to mice mono-colonized with *P*. *aeruginosa* (S1 Fig).

We previously demonstrated that neutropenia is sufficient for *P*. *aeruginosa* virulence [31], whereas both neutropenia and GI mucosal damage are required for *C*. *albicans* virulence [25]. Therefore, in the setting of neutropenia, 0% of the mice mono-colonized with *P*. *aeruginosa* survived (Fig 1B), with all deceased mice exhibiting evidence of extra-intestinal dissemination of *P*. *aeruginosa* (e.g. cultured *P*. *aeruginosa* from spleen homogenates) (Fig 1C). Surprisingly, 87.5–100% of mice co-colonized with *P*. *aeruginosa* and *C*. *albicans* survived after neutropenia (p<0.001 by Fisher’s exact, Fig 1B and 1C). There was no evidence of *C*. *albicans* dissemination (e.g. cultured *C*. *albicans* from liver homogenates) in any of the mice co-colonized with *C*. *albicans* and *P*. *aeruginosa* (Fig 1D).

To eliminate the potential immunomodulatory effects of other commensal gut microbiota, we repeated these experiments in germ-free mice. Again, although co-colonization with *C*. *albicans* did not significantly change *P*. *aeruginosa* GI colonization in germ-free mice (Fig 1E), co-colonization with *C*. *albicans* did increase length of survival (p = 0.04, log-rank test) and overall survival from *P*. *aeruginosa* infection (3 of 4 mice survived in the *C*. *albicans* and *P*. *aeruginosa* co-colonized group; 0 of 4 mice survived in the *P*. *aeruginosa* group; p = 0.07, Fisher’s exact) (Fig 1F). In deceased mice, *P*. *aeruginosa* dissemination was confirmed, but no evidence of *C*. *albicans* dissemination was noted (Fig 1G and 1H).

To exclude microbial strain-specific effects, we repeated these experiments with antibiotic-treated mice (n = 8) utilizing additional *P*. *aeruginosa* (PA14, PAK) and *C*. *albicans* strains (3153A, clinical isolate, biofilm[45]; Can098, clinical isolate, bloodstream; and Can091, clinical isolate, bloodstream) in all possible combinations. Again, *P*. *aeruginosa* GI colonization was unaffected by co-colonization with *C*. *albicans* (S2 Fig). *C*. *albicans* levels were also unaffected by *P*. *aeruginosa* (with the exception of decreased *C*. *albicans* 3153A levels in the presence of either *P*. *aeruginosa* PAO1 or PA14) (S2 Fig). Regardless of the *P*. *aeruginosa* or *C*. *albicans* strain used, mice co-colonized with *P*. *aeruginosa* and *C*. *albicans* had significantly increased length of survival (S3 Fig) and significantly increased overall survival (with the only exception being mice co-colonized with *P*. *aeruginosa* PA14 and *C*. *albicans* 3153A, p = 0.10, Fisher’s exact, Table 1) compared to mice mono-colonized with *P*. *aeruginosa*. All deceased mice exhibited evidence of *P*. *aeruginosa* dissemination (S3 Fig).

**Table 1:** Survival of C3H/HeN Mice GI Colonized with *P*. *aeruginosa* ± *C*. *albicans* after Induction of Neutropenia.

	No C. albicans	SC5314	Can091	Can098	3153A
**PAO1**	0/8	8/8 (p = 0.00008)^a^	7/8 (p = 0.0007)	5/8(p = 0.012)	7/8 (p = 0.0007)
**PA14**	0/8	8/8 (p = 0.00008)	6/8 (p = 0.0035)	6/8 (p = 0.0035)	3/8 (p = 0.10)
**PAK**	0/8	6/8 (p = 0.003)	4/8 (p = 0.038)	6/8 (p = 0.003)	4/8 (p = 0.038)

^a^ p-value by Fisher’s exact test compared to respective *P*. *aeruginosa* mono-colonized group.

In summary, we conclude that co-colonization with *C*. *albicans* does not affect *P*. *aeruginosa*’s ability to colonize the murine GI tract, regardless of the temporal sequence of colonization or the microbial strains used. Furthermore, co-colonization with *C*. *albicans* significantly increases both length of survival and overall survival from *P*. *aeruginosa* infection.

### *C*. *albicans* does not inhibit *Escherichia coli* GI colonization or virulence in mice

To determine whether *C*. *albicans*’s protective effect was unique to *P*. *aeruginosa*, we tested *Escherichia coli* (a clinical bloodstream isolate recovered from a pediatric cancer patient), another bacteria from the *Enterobacteriaceae* family frequently isolated from bloodstream infections in cancer patients [18,46]. As with *P*. *aeruginosa*, *C*. *albicans* did not affect *E*. *coli* GI colonization levels (S4 Fig). While *C*. *albicans* partially protected mice from *E*. *coli* mortality, this protective effect was not statistically significant (S4 Fig).

### The *C*. *albicans* quorum-sensing molecule farnesol does not affect *P*. *aeruginosa* GI colonization or virulence

Cross-kingdom cellular signaling has been documented between *P*. *aeruginosa* and *C*. *albicans in vitro*: the *P*. *aeruginosa* molecule 3-oxo-C12HSL affects *C*. *albicans* morphology [8], and the *C*. *albicans* metabolite farnesol inhibits *P*. *aeruginosa* quinolone signaling [11], reduces pyocyanin synthesis [11], and inhibits swarming motility [4]. Thus, we hypothesized that *C*. *albicans* farnesol might play a critical role in inhibiting *P*. *aeruginosa* virulence in our murine model. The deletion of the *C*. *albicans dpp3 gene*, which encodes a phosphatase that converts farnesyl pyrophosphate to farnesol [47], did not affect *P*. *aeruginosa* or *C*. *albicans* GI colonization levels (S5 Fig). Importantly, mice co-colonized with *C*. *albicans dpp3* ^Δ/Δ^ were still protected from *P*. *aeruginosa* (87.5% survival, 7 of 8 mice) compared to the *P*. *aeruginosa* mono-colonized group (0% survival, 0 of 8 mice) (S5 Fig). Finally, mice mono-colonized with *P*. *aeruginosa* and treated with oral farnesol [47] showed no significant difference in *P*. *aeruginosa* GI colonization levels and no significant difference in overall survival or length of survival from *P*. *aeruginosa* infection compared to untreated controls (S5 Fig). Thus, *C*. *albicans* farnesol does not inhibit *P*. *aeruginosa* virulence and is not responsible for the *C*. *albicans* protective effect from *P*. *aeruginosa* infection.

### *In vivo Pseudomonas aeruginosa* transcriptome analysis reveals *C*. *albicans* induced suppression of pyochelin and pyoverdine biosynthetic pathways

**Fig 2:**
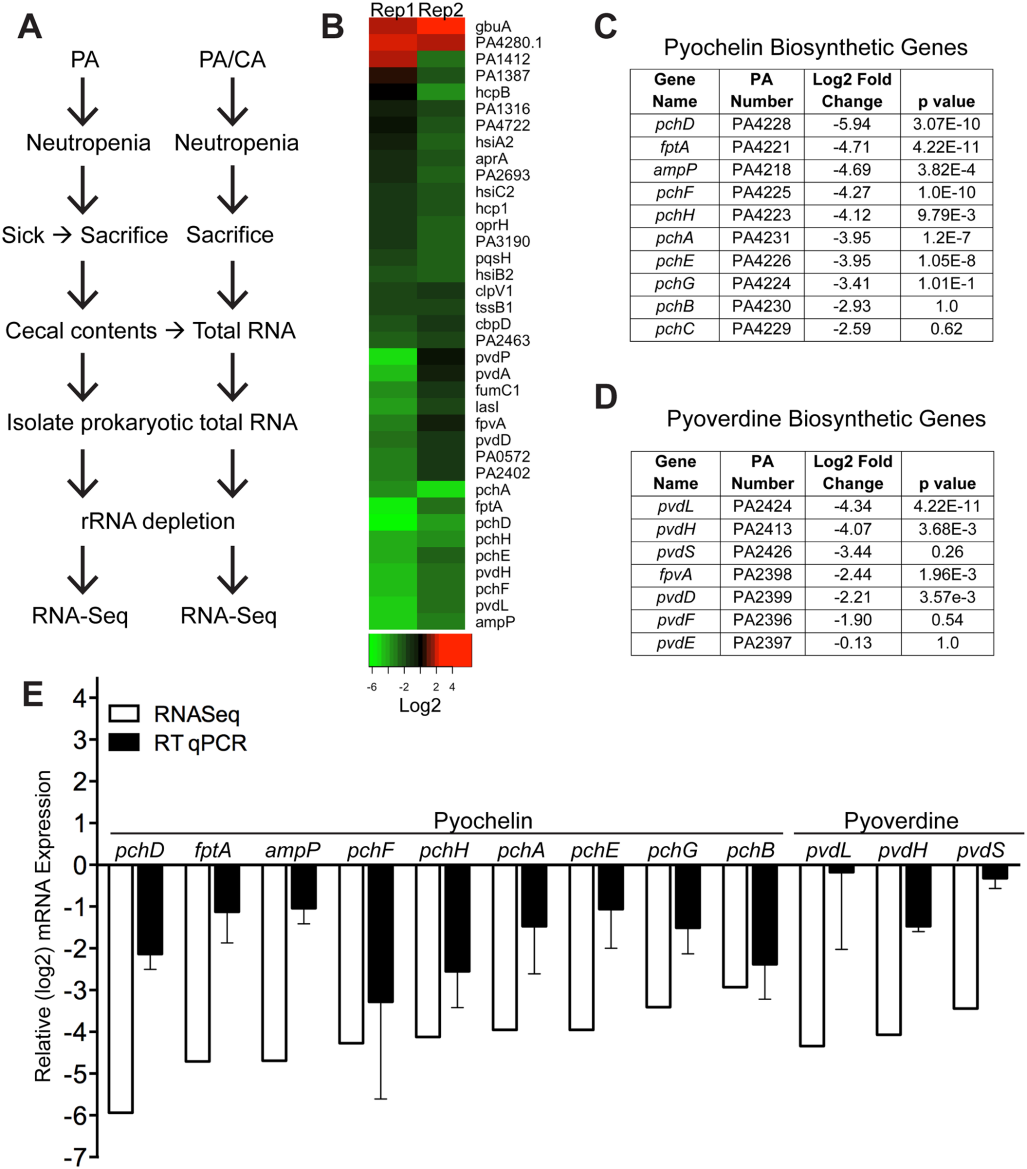
*C*. *albicans* suppresses pyochelin and pyoverdine biosynthetic pathways in *P*. *aeruginosa*. **A**) Experimental schema of *in vivo P*. *aeruginosa* transcriptome analysis experiments. PA, *P*. *aeruginosa*. CA, *C*. *albicans*. **B**) Heatmap of RNA-Seq analysis of *P*. *aeruginosa* strain PAO1 transcripts recovered from cecums of neutropenic mice co-colonized with *P*. *aeruginosa* and *C*. *albicans* compared to mice mono-colonized with *P*. *aeruginosa*. Prokaryotic mRNA from 8 mice pooled for each biological replicate; 2 biological replicates performed. RNA-Seq analysis performed with DESeq. **C**, **D**) Summary tables of *P*. *aeruginosa* pyochelin (**C**) and pyoverdine (**D**) gene expression (data from RNA-Seq analysis). **E**) RT qPCR of *P*. *aeruginosa* pyochelin and pyoverdine biosynthetic genes (performed on the same RNA samples used for RNA-Seq analysis). All data shown are means ± SEM. Assays were performed in triplicate. Statistical analysis was performed by unpaired Student’s *t-test*. * p< 0.05; ** p<0.01; ns, not significant.

We used RNA-Seq to examine the *in vivo* gene expression of *P*. *aeruginosa* PAO1 in the GI tract of neutropenic mice (n = 8), in the presence or absence of *C*. *albicans*. Our goal was to identify *P*. *aeruginosa* virulence genes normally activated during virulence from the GI tract (*P*. *aeruginosa* colonized mice) but suppressed by *C*. *albicans* in co-colonized mice (*P*. *aeruginosa* and *C*. *albicans* colonized mice) (Fig 2A). We utilized the antibiotic-treated, rather than germ-free, murine model to emulate the pathogenesis of *P*. *aeruginosa* infections in the clinical setting, realizing that we would be isolating transcripts from a complex mixed microbial population. We identified a total of 35 genes that were significantly repressed and only 2 genes whose mRNA levels were increased when *C*. *albicans* co-colonized the GI tract (Fig 2B, S1 File). 31% (11 of 35) of the down-regulated genes are known to be involved in pyochelin and pyoverdine biosynthetic pathways (Fig 2C and 2D). We performed RT qPCR of *P*. *aeruginosa* pyochelin and pyoverdine biosynthetic genes and confirmed a similar pattern of uniform repression of these genes consistent with our RNASeq results (Fig 2E). In sum, expression of *P*. *aeruginosa* pyochelin and pyoverdine genes significantly decreases in the murine GI tract when *C*. *albicans* is present.

### Deletion of both pyochelin and pyoverdine genes attenuates *P*. *aeruginosa* virulence

**Fig 3:**
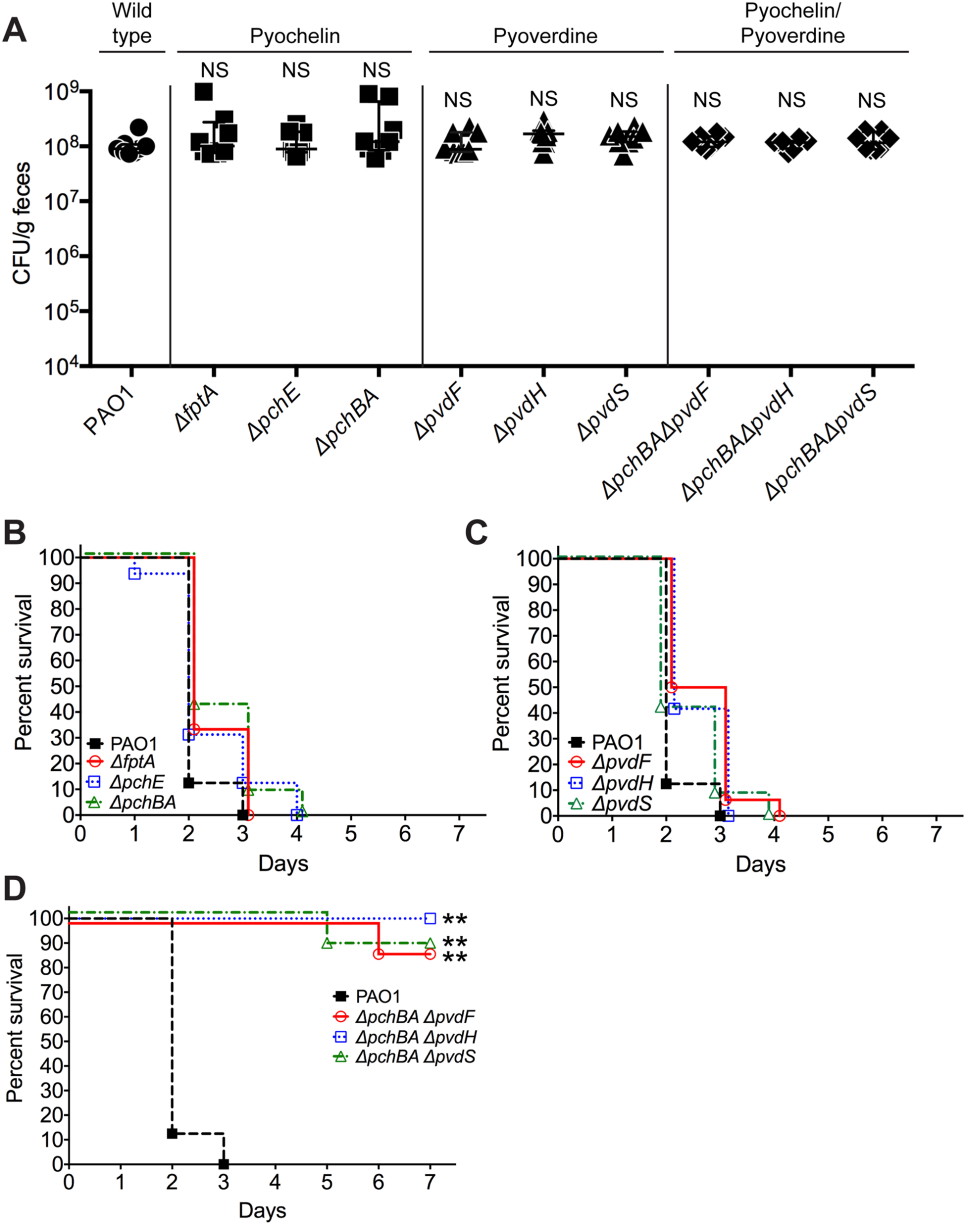
Deletion of *P*. *aeruginosa* pyochelin and pyoverdine genes attenuates *P*. *aeruginosa* virulence. **A**) GI colonization levels of wild type *P*. *aeruginosa* PAO1(circles); pyochelin deletional mutants (squares; Δ*fptA*, Δ*pchE*, Δ*pchBA*); pyoverdine deletional mutants (triangles; Δ*pvdF*, Δ*pvdH*, Δ*pvdS*); and pyochelin/pyoverdine deletional mutants (diamonds; Δ*pchBA*Δ*pvdF*, Δ*pchBA*Δ*pvdS*, Δ*pchBA*Δ*pvdH*) in C3H/HeN mice. n = 8 mice per group. Points represent results from individual animals. Horizontal lines with bars represent the median with interquartile range. Statistical analysis performed by Mann-Whitney test. * p< 0.05; ** p<0.01; ns, not significant. **B, C, D**) Survival curves of antibiotic-treated neutropenic C3H/HeN mice GI colonized with **B**) PAO1 pyochelin deletional mutants, **C**) PAO1 pyoverdine deletional mutants, and **D**) PAO1 pyochelin/pyoverdine deletional mutants. n = 8 mice per group. Statistical analysis performed by log-rank test. * p< 0.05; ** p<0.01; ns, not significant.

In order to assess the relative importance of specific pyochelin and/or pyoverdine genes for *P*. *aeruginosa* GI colonization or virulence, we constructed *P*. *aeruginosa* pyochelin, pyoverdine, and pyochelin/pyoverdine deletional mutants. Since iron is essential for microbial growth and survival, the deletion of pyochelin/pyoverdine genes might significantly limit *P*. *aeruginosa* iron acquisition and thus inhibit *P*. *aeruginosa* growth and GI colonization. Interestingly, deleting either pyochelin or pyoverdine genes, or both pyochelin and pyoverdine genes, did not affect *P*. *aeruginosa* GI colonization (Fig 3A).

In terms of virulence, deleting pyochelin (Fig 3B) or pyoverdine (Fig 3C) genes did not attenuate the virulence of *P*. *aeruginosa*, but deleting both pyochelin and pyoverdine genes significantly increased survival from *P*. *aeruginosa* infection (87.5–100% survival, p = <0.0001, Fisher’s exact test) when compared to mice colonized with wild-type *P*. *aeruginosa* (0% survival) (Fig 3D). Complementation with the pyochelin genes *pchBA* (pME6477) in the pyochelin/pyoverdine deletional mutants was sufficient to restore *P*. *aeruginosa* virulence (S6 Fig) thus proving that the deletion phenotype was not due to unexpected “off-target” effects of deletion. *P*. *aeruginosa* dissemination was confirmed by the presence of cultured *P*. *aeruginosa* or *P*. *aeruginosa* mutants from spleen homogenates of deceased mice (S6 Fig). Thus, either *P*. *aeruginosa* pyochelin or pyoverdine is sufficient to maintain *P*. *aeruginosa* virulence.

### Addition of *C*. *albicans* to a *P*. *aeruginosa*-colonized murine gut does not increase gut iron levels

**Fig 4:**
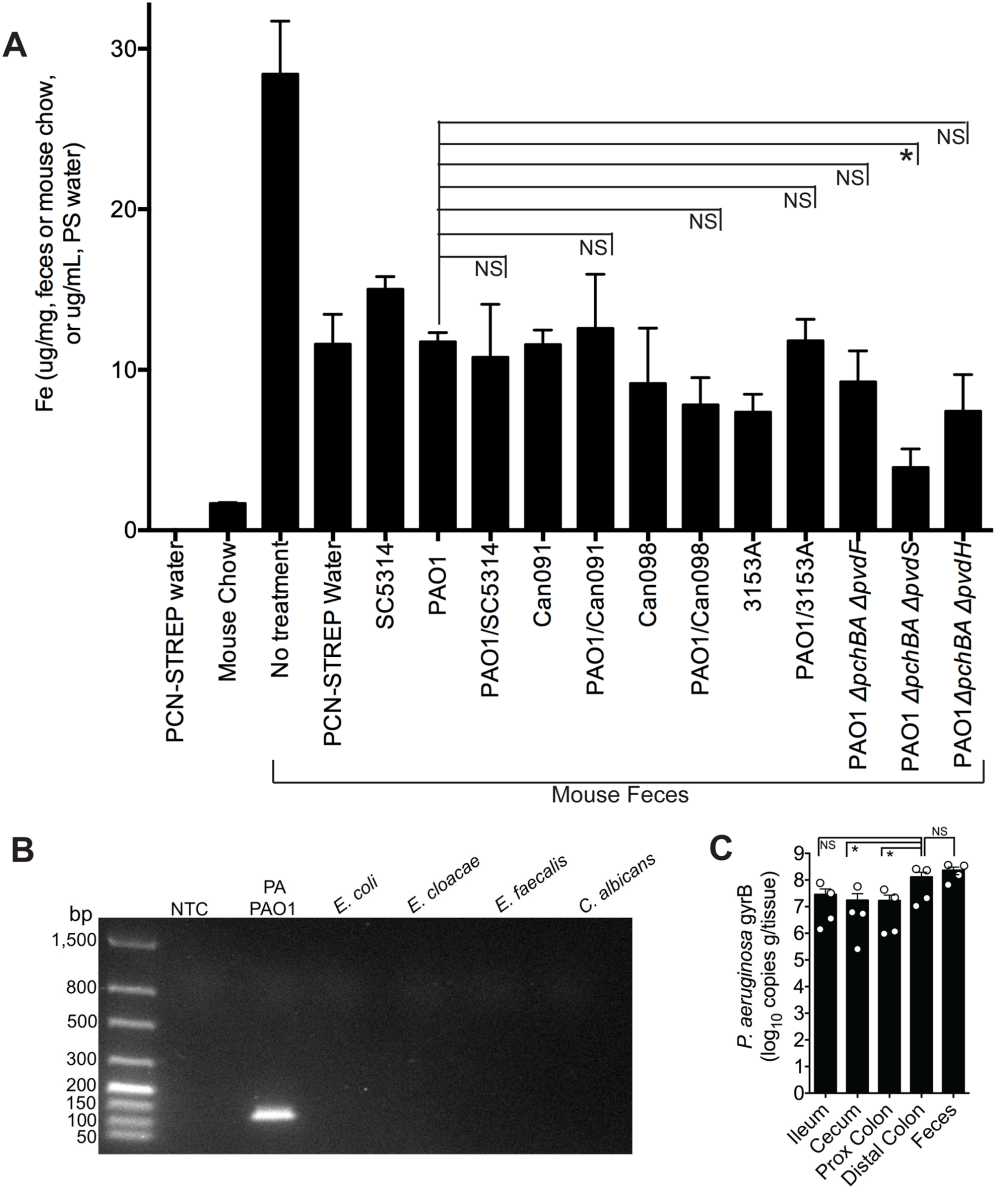
The addition of *C*. *albicans* to a *P*. *aeruginosa*-colonized murine gut does not significantly increase gut iron levels. **A**) Total iron content of antibiotic water (penicillin/streptomycin), mouse chow (Teklad Global 16% Protein Rodent Diet, Harlan), and fecal specimens of C3H/HeN mice treated ± antibiotics and colonized ± *P*. *aeruginosa* and/or *C*. *albicans*. Total iron content (Fe^2+^and Fe^3+^) was determined by ferrozine assay [48]. All data shown are means ± SEM. Assays for mouse chow and antibiotic water were performed in triplicate. For fecal measurements, feces from individual mice (n = 4 mice per group) were analyzed. Statistical analysis was performed by Mann-Whitney test. * p< 0.05; ** p<0.01; ns, not significant. **B**) DNA gel electrophoresis of *P*. *aeruginosa gyrB* qPCR products generated using gDNA (100 ng) from *P*. *aeruginosa* PAO1, *Escherichia coli* ATCC10798, *Enterobacter cloacae* (clinical isolate), *Enterococcus faecalis* (clinical isolate), *C*. *albicans* SC5314, and a no template control (NTC). The predicted size of the *P*. *aeruginosa* gyrB qPCR product is 131 bp. **C**) *P*. *aeruginosa gyrB* levels (expressed as copies per gram/tissue) in feces and washed intestinal segments (2 cm sections; ileum; cecum, proximal colon and distal colon) of antibiotic-treated C3H/HeN mice mono-colonized with *P*. *aeruginosa* strain PAO1. Bars shown are means ± SEM. n = 4 mice per group. Points represent results from individual animals. Statistical analysis was performed by Mann-Whitney test. * p< 0.05; ** p<0.01; ns, not significant.

Since it is well-established that iron can repress the synthesis of *P*. *aeruginosa* siderophores, we first determined whether the iron concentration in the GI tract (as determined by measuring total fecal iron content) of a mouse co-colonized with *P*. *aeruginosa* and *C*. *albicans* was higher than in mice mono-colonized with *P*. *aeruginosa*, as this would then explain the observed decrease in *P*. *aeruginosa* pyochelin and pyoverdine gene expression when *C*. *albicans* is present. Total fecal iron content (Fe^2+^and Fe^3+^ as determined by a ferrozine assay [48]) in untreated (no antibiotics, no microbes) mice was greater than in antibiotic treated mice (Fig 4A). Mouse chow did have detectable iron (albeit a very low concentration), whereas the antibiotic (penicillin-streptomycin) water did not have any detectable iron (Fig 4A). In general, introduction of *P*. *aeruginosa* and/or *C*. *albicans* into the GI tract of antibiotic-treated mice did not appreciably increase fecal iron levels compared to mice treated only with antibiotics. But most importantly, fecal iron levels in mice co-colonized with *P*. *aeruginosa* and *C*. *albicans* were not significantly increased compared to mice mono-colonized with the corresponding *P*. *aeruginosa* strain (e.g. fecal iron levels in mouse co-colonized with PAO1 and SC5314 were not significantly higher than in mice mono-colonized with PAO1) (Fig 4A). Furthermore, mice colonized with *P*. *aeruginosa* pyochelin and pyoverdine mutants did not have significantly increased fecal iron levels compared to mice colonized with wild-type PAO1 (Fig 4A). Mice colonized with the pyochelin/pyoverdine deletional mutant PAO1Δ*pchBA*Δ*pvdS*, however, did have significantly lower fecal iron levels compared to PAO1, which may be due to an a unique interaction with the gut microbiota or host.

Iron is critical for *P*. *aeruginosa* biofilm initiation and maturation [49–51]. Furthermore, disruptions in *P*. *aeruginosa* iron metabolism have been shown to inhibit *P*. *aeruginosa* biofilm formation [52]. This point is particularly relevant given that there are at least two distinct populations of *P*. *aeruginosa* in the murine GI tract: *P*. *aeruginosa* in the gut lumen and *P*. *aeruginosa* adherent to the GI epithelium, a subset of which may be in biofilms. Fecal specimens are routinely used to measure gut microbial abundance or profile gut microbial populations, but these data may better represent gut lumen microbial populations and may underrepresent microbial populations adherent to GI epithelium. Therefore in order to determine whether measuring *P*. *aeruginosa* levels in the feces was an accurate surrogate for the total *P*. *aeruginosa* levels in the GI tract, we utilized a *P*. *aeruginosa*-specific qPCR assay to measure copies of the *P*. *aeruginosa gyrB* gene from gDNA extracted from feces and gastrointestinal segments (flushed and rinsed with PBS to remove the luminal contents) (Fig 4B)—rather than culturing intestinal tissue homogenates for *P*. *aeruginosa* which might miss (or underrepresent) *P*. *aeruginosa* present biofilms adherent to intestinal tissue. Total *P*. *aeruginosa* levels (as represented by copies of *P*. *aeruginosa gyrB* per gram/tissue) in the feces were not significantly different from *P*. *aeruginosa* levels in the distal colon (Fig 4C), suggesting that measuring *P*. *aeruginosa* in feces is an adequate surrogate for total *P*. *aeruginosa* in the distal colon. Of note, *P*. *aeruginosa* levels in the more proximal intestinal segments were approximately one log lower than in the distal colon (Fig 4C).

Thus, the addition of *C*. *albicans* to a *P*. *aeruginosa*-colonized murine GI tract does not increase fecal iron levels. Therefore, iron is unlikely to cause the *C*. *albicans*-induced decrease in *P*. *aeruginosa* pyochelin and pyoverdine gene expression.

### *C*. *albicans* secreted factors inhibit *P*. *aeruginosa* pyochelin and pyoverdine gene expression and pyoverdine production *in vitro*

**Fig 5:**
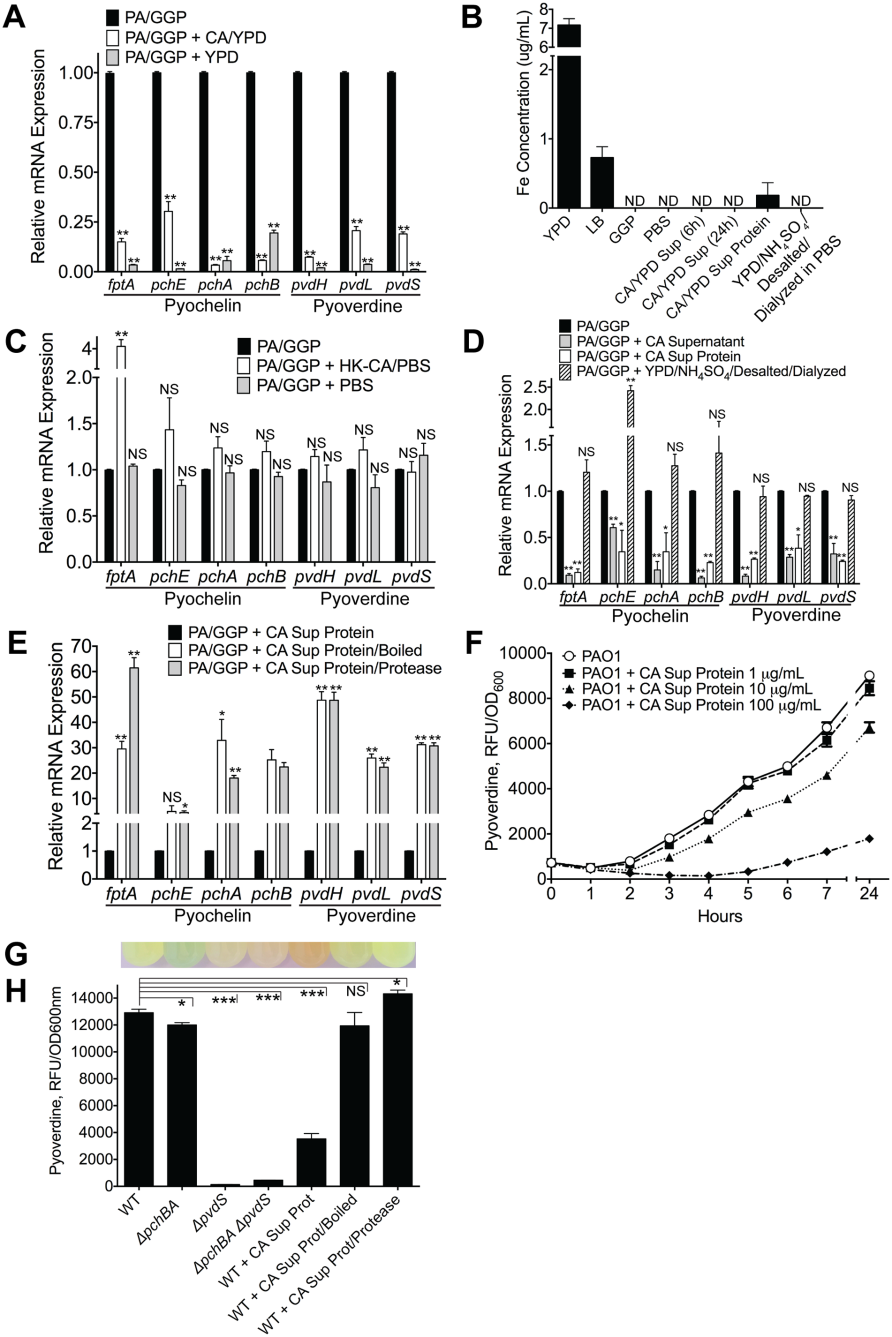
*C*. *albicans* secreted factors inhibit *P*. *aeruginosa* pyochelin and pyoverdine gene expression and pyoverdine production *in vitro*. **A)** Pyochelin and pyoverdine gene expression by RT qPCR of *P*. *aeruginosa* PAO1 grown *in vitro* to mid-log phase in iron-limited GGP media ± live *C*. *albicans* (grown in YPD media) or YPD media alone control. *P*. *aeruginosa* and *C*. *albicans* co-cultures were mixed in a 1:1 ratio and co-incubated at 37°C for 10 minutes before RNA isolation. **B)** Total iron content of media (YPD, LB, GGP, PBS), supernatant of *C*. *albicans* cultures grown in YPD, and *C*. *albicans* supernatant protein preparations (pre and post iron depletion). Total iron content (Fe^2+^and Fe^3+^) was determined by ferrozine assay [48]. All data shown are means ± SEM. Assays were performed in triplicate. Statistical analysis was performed by unpaired Student’s *t-test*. **C, D, E**) Pyochelin and pyoverdine gene expression by RT qPCR of *P*. *aeruginosa* PAO1 grown *in vitro* to mid-log phase in iron-limited GGP media with or without the following: **C)** Heat-killed (HK) *C*. *albicans* cells (suspended in PBS) or PBS alone control. **D)** *C*. *albicans* supernatant (stationary cultures grown in YPD at 30°C), *C*. *albicans* supernatant protein (supernatants of *C*. *albicans* stationary cultures grown in YPD; precipitated with ammonium sulfate, desalted, and dialyzed against PBS precipitation; final concentration of 100 μg/mL), and YPD subjected to the same protein purification process as *C*. *albicans* supernatant proteins. **E)** *C*. *albicans* supernatant proteins ± physical (boiled for 60 minutes) or chemical (treated with protease from *Streptomyces griseus* for 60 minutes) denaturation. HK *C*. *albicans* cells, PBS, and *C*. *albicans* supernatant were mixed in a 1:1 ratio with live *P*. *aeruginosa* culture. *C*. *albicans* supernatant protein was added to a final concentration of 100 ug/mL. All co-cultures were incubated at 37°C for 10 minutes before RNA isolation. All data shown for pyoverdine and pyochelin RT qPCR assay (Fig 5A, 5C, 5D and 5E) are means ± SEM. Assays were performed in triplicate. Statistical analysis was performed by unpaired Student’s *t-test*. **F)** Dose dependent effect of *C*. *albicans* supernatant protein on pyoverdine production (as determined by measuring fluorescence at 400±10/460±40 nm excitation/emission and normalizing to cell density measured at 600 nm) by *P*. *aeruginosa* PAO1 grown in GGP media at 37°C over 24 hours. **G, H**) Representative picture of (**G**) and pyoverdine levels of (**H**) a wild-type PAO1 stationary culture (after 24 hours of growth in GGP media) compared to cultures of pyochelin deletional mutant PAO1Δ*pchBA*, pyoverdine deletional mutant PAO1Δ*pvdS*, pyochelin/pyoverdine double mutant PAO1Δ*pchBA* Δ*pvdS*, PAO1 ± *C*. *albicans* SC5314 supernatant protein (final concentration 100 ug/mL), PAO1 ± *C*. *albicans* SC5314 supernatant protein boiled, and PAO1 ± *C*. *albicans* SC5314 supernatant protein treated with *Streptomyces griseus* protease. Bars shown are the means ± SEM. Assays were performed in triplicate. Statistical analysis performed by unpaired Student’s *t-test*, * p< 0.05; ** p<0.01, *** p<0.0001; ns, not significant.

We next assessed whether *C*. *albicans* inhibits *P*. *aeruginosa* pyochelin and pyoverdine gene expression through a direct mechanism. We grew *P*. *aeruginosa* in GGP medium (a medium with limited iron availability to enhance the production of pyochelin and pyoverdine) to mid-log phase, added either mid-log phase *C*. *albicans* grown in YPD medium or a YPD alone control, co-incubated at 37°C for 10 minutes, and then isolated bacterial RNA. Pyochelin and pyoverdine gene expression uniformly decreased following addition of *C*. *albicans* (yeast or hyphal form), compared to a *P*. *aeruginosa* only control (Fig 5A, S7 Fig). The YPD only control, however, also significantly inhibited pyochelin and pyoverdine gene expression. This was most likely due to the relatively high iron concentration of YPD compared GGP medium (Fig 5B). To confirm this hypothesis, we repeated the pyochelin and pyoverdine gene expression experiments using YPD, YPD that had been depleted of iron, using the chelating agent Chelex100, and iron-depleted YPD supplemented with iron (to iron concentrations comparable to untreated YPD) (S8 Fig). The results confirmed that the iron in YPD was responsible for the inhibition of pyochelin and pyoverdine gene expression (S8 Fig).

We were, however, unable to detect any iron in YPD in which *C*. *albicans* had been grown to mid-log phase, presumably because the iron had been taken up by the yeast cells (Fig 5B). Thus, to further investigate whether this effect was from direct *C*. *albicans* and *P*. *aeruginosa* interactions, we exposed mid-log phase *P*. *aeruginosa* grown in GGP to heat-killed (HK) *C*. *albicans* cells suspended in PBS or a PBS only control. Addition of HK *C*. *albicans* or the PBS control did not suppress pyochelin and/or pyoverdine gene expression (Fig 5C).

We then asked whether a *C*. *albicans* secreted factor might be mediating the *P*. *aeruginosa* pyochelin and pyoverdine gene expression suppression. Supernatant from a *C*. *albicans* overnight culture (YPD, 30°C) significantly decreased *P*. *aeruginosa* pyochelin and pyoverdine gene expression (Fig 5D), and iron was not detectable in the *C*. *albicans* YPD supernatant (Fig 5B). To further determine if this inhibitory *C*. *albicans* secreted factor was a protein, we isolated *C*. *albicans* supernatant proteins by ammonium sulfate precipitation, followed by desalting with Sephadex G-25 gel filtration, and then dialysis against PBS. Indeed, *C*. *albicans* supernatant proteins alone significantly inhibited *P*. *aeruginosa* pyochelin and pyoverdine gene expression *in vitro* (Fig 5D). Iron was detected in the *C*. *albicans* supernatant protein, albeit at very low concentrations (mean 0.1830 ug/mL), but we were able to deplete the iron in the *C*. *albicans* supernatant protein to undetectable levels (Fig 5B). YPD that had undergone the same protein purification process (ammonium sulfate precipitation, desalting, and dialysis) had no detectable iron and did not significantly decrease *P*. *aeruginosa* pyochelin and pyoverdine gene expression (Fig 5B and 5D).

To further delineate whether the *C*. *albicans* secreted factor mediating this effect was a protein, we subjected the *C*. *albicans* supernatant protein to physical (boiled for 60 minutes) or chemical (treated with protease from *Streptomyces griseus* for 60 minutes) denaturation. Addition of boiled or protease-treated *C*. *albicans* supernatant protein to *P*. *aeruginosa* cultures resulted in significantly higher *P*. *aeruginosa* pyochelin and pyoverdine gene expression compared to *P*. *aeruginosa* cultures given untreated *C*. *albicans* supernatant protein (Fig 5E), strongly suggesting that the inhibitory *C*. *albicans* secreted effector is a protein.

In order to corroborate our gene expression data with protein-level expression, we utilized a well-established method of quantifying pyoverdine levels, by measuring the characteristic green fluorescence attributed to pyoverdine (Fig 5G). We first demonstrated that *P*. *aeruginosa* pyoverdine production was inhibited by *C*. *albicans* secreted protein in a dose-dependent manner (Fig 5F), comparable to levels in a *P*. *aeruginosa* pyoverdine deficient mutant (Fig 5G and 5H). Consistent with our gene expression data, *C*. *albicans* supernatant proteins inhibited pyoverdine production, and physical or chemical denaturation of *C*. *albicans* secreted protein abrogated this effect (Fig 5G and 5H). *C*. *albicans* secreted proteins did not inhibit *P*. *aeruginosa* growth in GGP medium *in vitro* (S9 Fig). Finally, we found comparable results (S10 Fig) when repeating these experiments using the additional *P*. *aeruginosa* and *C*. *albicans* strains used in in the *in vivo* experiments described previously (S2 and S3 Figs).

In sum, a *C*. *albicans* secreted factor, most likely a secreted protein, suppresses *P*. *aeruginosa* pyoverdine and pyochelin gene expression and reduces pyoverdine production *in vitro*.

### *C*. *albicans* secreted proteins inhibit *P*. *aeruginosa* mediated cytotoxicity *in vitro* and inhibit *P*. *aeruginosa* virulence *in vivo*

The relationship between iron acquisition and microbial virulence is well-established. Iron uptake mutants can be avirulent [53], and iron deficiency is associated with increased resistance to infection [54]. Suppression of iron acquisition could lead to decreased growth and colonization and thus decreased risk for virulence. In fact, high-level gut bacterial colonization significantly increases the risk for virulence from the gut in cancer patients [29]. In our study, however, inhibition or deletion of pyochelin and pyoverdine had no effect on *P*. *aeruginosa* GI colonization.

**Fig 6:**
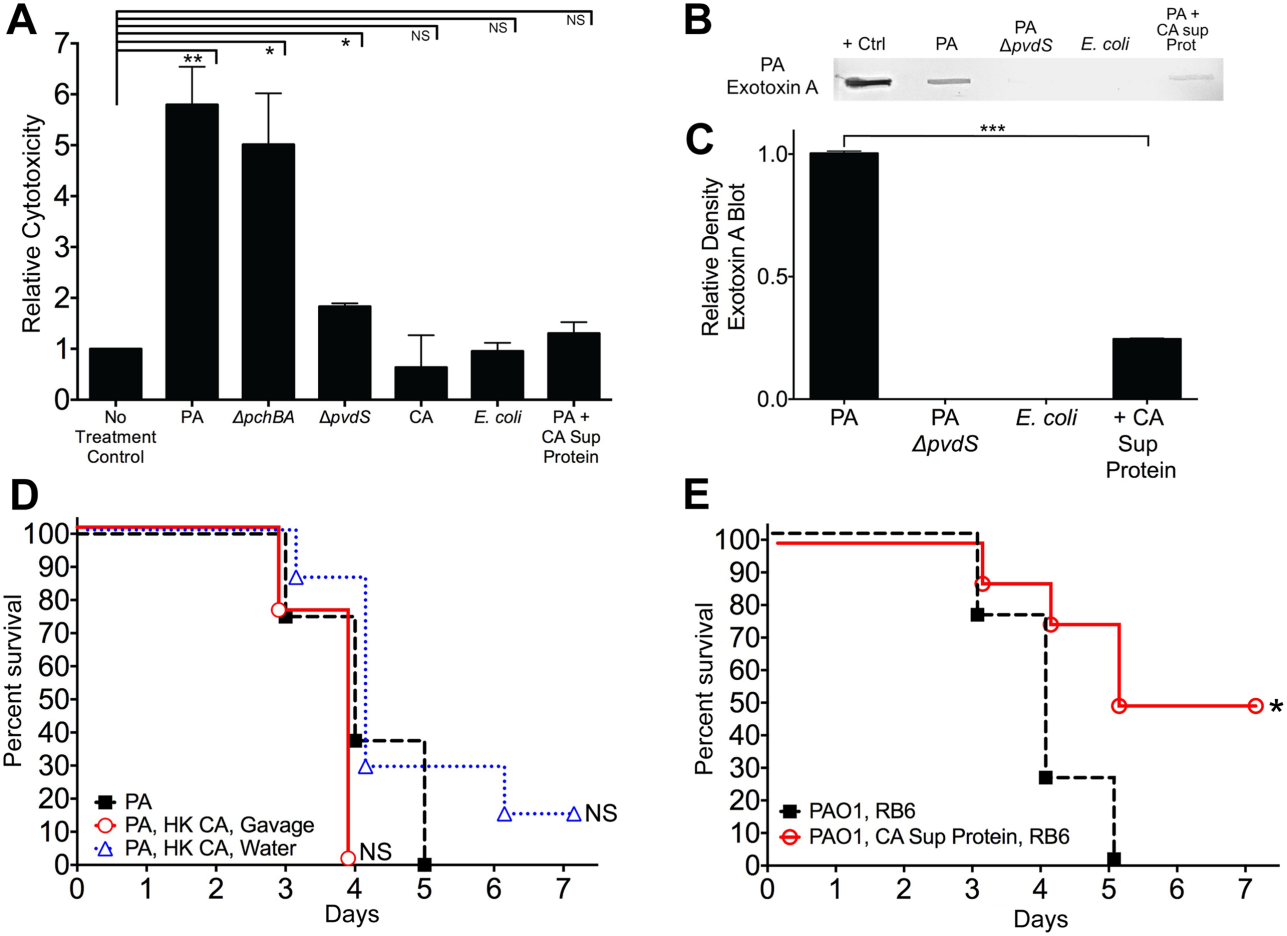
*C*. *albicans* secreted factors inhibit *P*. *aeruginosa* mediated cytotoxicity *in vitro*. **A**) Protective effect of *C*. *albicans* secreted protein on *P*. *aeruginosa* supernatant protein-induced cytotoxicity. Human colonocytes, HT-29, were exposed to *in vitro* microbial culture supernatant proteins (1 μg/uL) for 3 hours. Cell toxicity was measured by CytoTox-Glo assay (Promega). Microbial culture supernatant proteins were isolated from overnight stationary cultures of wild type PAO1, pyochelin deletional mutant PAO1Δ*pchBA*, pyoverdine deletional mutant PAO1Δ*pvdS*, *C*. *albicans* SC5314, *E*. *coli* ATCC 10798, PAO1 grown with *C*. *albicans* SC5314 supernatant protein (final concentration 100 ug/mL). Bars shown are the means ± SEM. Assays were performed in triplicate. Statistical analysis performed by unpaired Student’s *t-test*, * p< 0.05; ** p<0.01, *** p<0.0001; ns, not significant. **B**) Representative *P*. *aeruginosa* Exotoxin A immunoblot assay of culture supernatant proteins (250 μg) from wild-type PAO1, PAO1Δ*pvdS*, *E*. *coli* ATCC 10798, wild-type PAO1 grown with *C*. *albicans* secreted protein (100 μg/mL), and *C*. *albicans* strain SC5314. The primary antibody used was a polyclonal rabbit IgG to *P*. *aeruginosa* exotoxin A (Sigma, P2318). **C**) Relative density of Exotoxin A immunoblot as measured by ImageJ Software. Bars shown are the means ± SEM. Assays were performed in triplicate. Statistical analysis performed by unpaired Student’s *t-test*, * p< 0.05; ** p<0.01, *** p<0.0001; ns, not significant. **D, E**) Survival curves of antibiotic-treated neutropenic C3H/HeN mice GI colonized with *P*. *aeruginosa* PAO1 strain and treated ± with (**D**) heat-killed (HK) *C*. *albicans* cells by oral gavage or suspended in the drinking water or (**E**) iron depleted *C*. *albicans* secreted proteins (200 ug by oral gavage daily and 100 μg/mL suspended in the drinking water, refreshed every 2 days). n = 8 mice per group. Survival curves analyzed by log-rank test. * p< 0.05; ** p<0.01; ns, not significant.

Interestingly, pyoverdine is not only a siderophore but also a signaling molecule that induces the production of two extracellular virulence factors, the protease PrpL and exotoxin A [55]. Extracellular virulence factors (including proteases, cytotoxins, and phospholipases) have been shown to contribute to *P*. *aeruginosa* virulence in various animal models [56–58]. Therefore, we postulated that *C*. *albicans* mediated protection from *P*. *aeruginosa* infection might be due to inhibition of *P*. *aeruginosa* extracellular cytotoxic molecule production (i.e. exotoxin A, PrpL, etc). To explore this hypothesis, we utilized an *in vitro* cytotoxicity assay using cultured human colonocytes and *P*. *aeruginosa* culture supernatant proteins (recovered from stationary-phase cultures grown in iron-limited GGP media). *P*. *aeruginosa* culture supernatant proteins had variable levels of cytotoxicity against cultured human colonocytes: wild-type *P*. *aeruginosa* (5.8 fold increase compared to untreated control) > Δ*pchBA* (5.0) > Δ*pvdS* (1.8) > wild-type *P*. *aeruginosa* + *C*. *albicans* secreted protein (1.3) > *E*. *coli* secreted protein (0.95) > *C*. *albicans* secreted protein (0.6) (Fig 6A). In fact, the cytotoxicity induced by supernatant proteins recovered from *P*. *aeruginosa* grown with *C*. *albicans* secreted proteins was not significantly different than the untreated control group. Finally, immunoblotting showed that exotoxin A was nearly 80% reduced when *P*. *aeruginosa* was grown with *C*. *albicans* secreted proteins (Fig 6B and 6C). In summary, *C*. *albicans* secreted proteins inhibit the production of *P*. *aeruginosa* extracellular cytotoxic molecules, such as exotoxin A, and thus inhibit the cytotoxic effect of *P*. *aeruginosa* extracellular proteins against cultured human colonocytes.

To determine whether live *C*. *albicans* was necessary to protect against invasive *P*. *aeruginosa in vivo*, we first established *P*. *aeruginosa* mono-colonization in antibiotic-treated mice. We then treated *P*. *aeruginosa* mono-colonized mice with heat-killed (HK) *C*. *albicans* cells by gavage; HK *C*. *albicans* suspended in drinking water; iron-depleted *C*. *albicans* supernatant protein (200 ug by oral gavage daily and 100 μg/mL suspended in drinking water, renewed every 2 days); or a no treatment control on the day prior to induction of neutropenia and maintained throughout the duration of the experiment. Mice treated with HK *C*. *albicans* were not protected from *P*. *aeruginosa* infection (Fig 6D), while mice treated with iron-depleted *C*. *albicans* supernatant protein had significantly increased length of survival (p = 0.0208, log-rank test, Fig 6E) and overall survival from *P*. *aeruginosa* infection (4 of 8 mice, p = 0.038, Fisher’s exact test). Oral delivery of protein and peptide drugs is often problematic because of the high acidity of the stomach and proteolysis by intestinal enzymes. Yet both nutritional and pharmaceutical proteins can survive passage through the GI tract, albeit in minute quantities[59,60]. Given that laboratory mice drink ~4–8 ml water/day (with C3H mice drinking ~ 5–6 ml/day) [61], the mice used in this experiment would have ingested up to 800 ug of *C*. *albicans* supernatant protein per day. Furthermore, the total GI transit time in mice is roughly 6 hours compared to between 40–50 hours in humans [62]. Therefore, this rapid transit time may help proteins better survive gastric acidity and intestinal proteolysis, and thus be delivered throughout the murine GI tract. Despite the difficulty of surviving passage through the stomach and intestine, the *C*. *albicans* supernatant proteins that survived intestinal passage still afforded some degree of protection from *P*. *aeruginosa* infection, although not to the degree seen with *C*. *albicans* co-colonization. These data suggest that a *C*. *albicans* secretable factor, most likely a secreted protein, is responsible for *C*. *albicans*-mediated protection from *P*. *aeruginosa* infection in our murine model.

### Iron supplementation restores *P*. *aeruginosa* virulence in *P*. *aeruginosa*-*C*. *albicans* co-colonized mice

**Fig 7:**
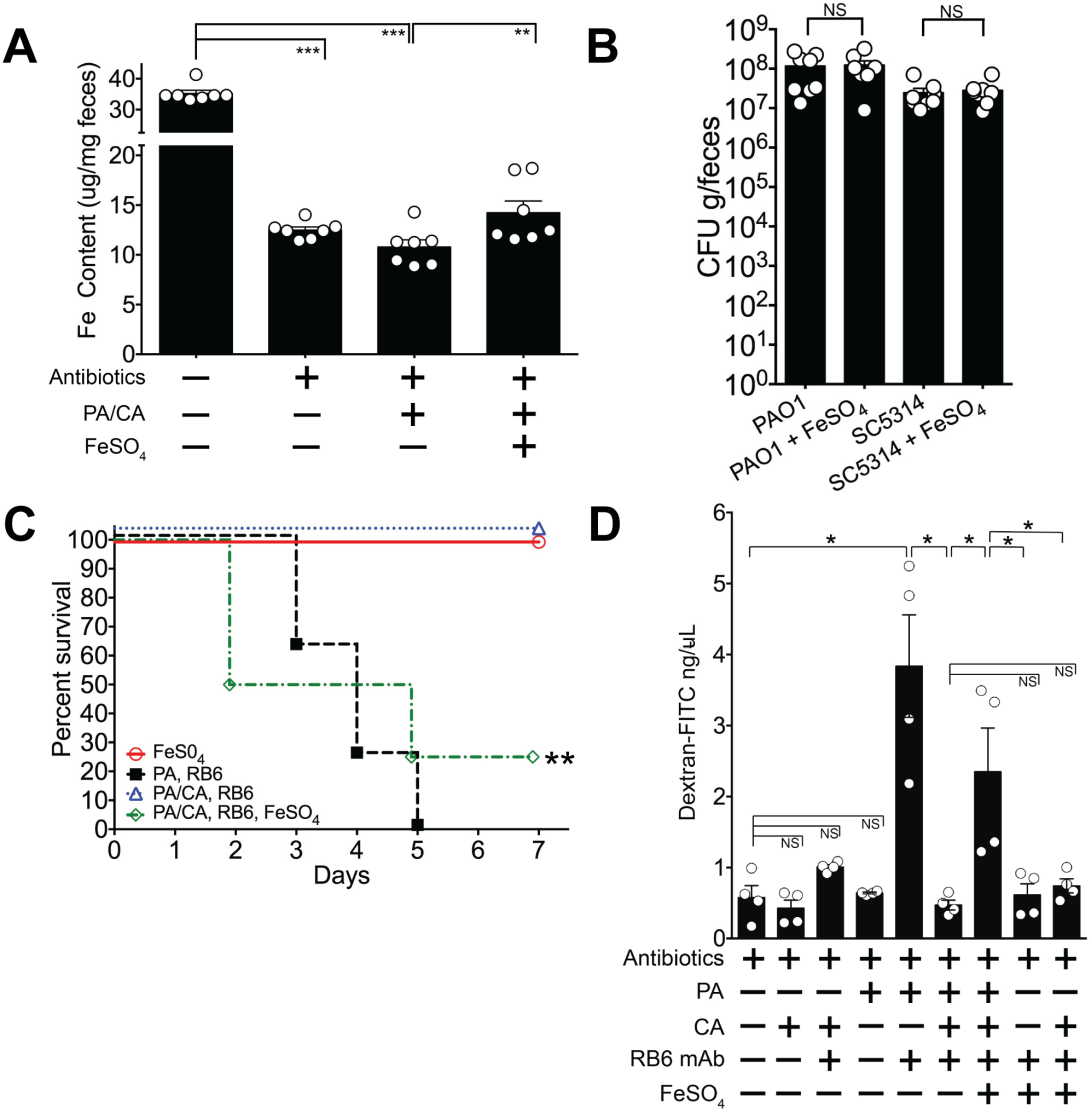
Iron supplementation restores *P*. *aeruginosa* virulence in *P*. *aeruginosa* and *C*. *albicans* colonized mice. **A**) Oral FeSO_4_ increases fecal total iron levels (as assessed by ferrozine assay) in C3H/HeN mice treated with oral antibiotics (penicillin, streptomycin) and then co-colonized with *P*. *aeruginosa* PAO1 and *C*. *albicans* SC5314. Bars shown are means ± SEM. n = 7 mice per group. Points represent results from individual animals. Statistical analysis was performed by Mann-Whitney test. * p< 0.05; ** p<0.01; ns, not significant. PA, *P*. *aeruginosa*. CA, *C*. *albicans*. **B)** *P*. *aeruginosa* PAO1 and *C*. *albicans* SC5314 GI colonization levels in antibiotic-treated adult mice (C3H/HeN) ± FeSO_4_ treatment. Mice treated with FeSO_4_ were orally gavaged 0.1 ml of 40 mM FeSO_4_ for 7 days. Microbial GI colonization levels were checked 7 days after FeSO_4_ treatment. n = 8 mice per group. Points represent results from individual animals. Horizontal lines with bars represent the median with interquartile range. Statistical analysis performed by Mann-Whitney test. * p< 0.05; ** p<0.01; ns, not significant. **C)** Oral FeSO_4_ restores *P*. *aeruginosa* virulence in neutropenic C3H/HeN mice co-colonized with *P*. *aeruginosa* PAO1 and *C*. *albicans* SC5314. n = 8 mice per group. Survival curves analyzed by log-rank test. * p< 0.05; ** p<0.01; ns, not significant. RB6, RB6-8C5 mAb. **D**) Intestinal permeability assay of mice. Mice were treated with antibiotics (penicillin-streptomycin); colonized with *P*. *aeruginosa* PAO1 and/or *C*. *albicans* SC5314; injected with RB6-8C5 monoclonal antibody to induce neutropenia; and/or treated with treated with FeSO_4_ (orally gavaged 0.1 ml of 40 mM FeSO_4_ for 5 days). Mice were then fasted for 4.5 hours, gavaged with FITC-Dextran (FD4, Sigma); and serum collected 3 hours later. n = 4 mice per group. Bars shown are means + SEM. Points represent results from individual animals. Statistical analysis performed by Mann-Whitney test. * p< 0.05; ** p<0.01; ns, not significant.

*P*. *aeruginosa* has an alternative iron uptake system, the FeoABC system, that allows uptake of Fe^2+^ which is soluble and present in anaerobic conditions or in microaerobic environments at lower pH [63]. The FeoABC system is active during infections in patients with cystic fibrosis [64,65]. Since the distal gut is an anaerobic/ microaerobic environment with lower pH [66], we hypothesized that if supplemental oral iron were introduced into the GI tract of *P*. *aeruginosa* and *C*. *albicans* colonized mice, *P*. *aeruginosa* might utilize the FeoABC system to acquire iron and thus restore *P*. *aeruginosa* virulence. Consistent with our prior findings (Fig 4), total fecal iron content (Fe^2+^and Fe^3+^ as determined by a ferrozine assay [48]) in antibiotic treated mice was significantly lower than in untreated mice (Fig 7A). Introduction of *P*. *aeruginosa* and *C*. *albicans* into the GI tract of antibiotic-treated mice did not appreciably increase fecal iron levels. Oral administration of FeSO_4_, however, significantly increased overall fecal iron levels in *P*. *aeruginosa*—*C*. *albicans* co-colonized mice (Fig 7A) but did not restore Fe levels to those found in untreated mice. FeSO_4_ supplementation did not change *P*. *aeruginosa* or *C*. *albicans* GI colonization levels when compared to the untreated control group (Fig 7B). Importantly, iron supplementation restored *P*. *aeruginosa* virulence: 25% survival from *P*. *aeruginosa* infection in *P*. *aeruginosa* and *C*. *albicans* co-colonized mice treated with FeSO_4_ compared to 100% survival in untreated *P*. *aeruginosa* and *C*. *albicans* co-colonized mice (overall survival: p = 0.003, Fisher’s exact test; length of survival: p = 0.0024, log-rank test) (Fig 7C).

While iron supplementation clearly restored *P*. *aeruginosa* virulence, we were still uncertain as to why. To best answer this question, we turned our focus to the three primary defense mechanisms that prevent microbial translocation from the GI tract in humans and mice: 1) gut microbiota homeostasis, preventing the overgrowth or expansion of pathogenic microbes; 2) intact cellular immunity, particularly neutrophils; and 3) intact intestinal mucosal barriers [21–23]. Iron supplementation did not promote *P*. *aeruginosa* expansion in the gut (Fig 7B), so increased bacterial burden was not the cause of increased virulence. Excess iron may inhibit host phagocytic cells, repressing both chemotaxis and phagocytosis [67,68]. However, in our murine model, absolute neutrophil and macrophage counts are already < 100 cells/mm^3^ [31], so it is unlikely that further qualitative immune deficits (chemotactic or phagocytic) would have significant consequences. The final possibility was that iron may have some role in modulating gut epithelial integrity—either directly through the host or indirectly through its effect on *P*. *aeruginosa*. To investigate this possibility, we used an intestinal permeability assay in which we orally gavaged mice with fluorescent-labeled dextran (FITC-dextran) and then measured FITC-dextran levels in the serum[69]. Low levels of serum FITC-dextran would indicate low intestinal permeability (strong gut epithelial integrity) and thus low absorption from the gut. Mono-colonization with *P*. *aeruginosa*, or mono-colonization with *C*. *albicans* did not significantly increase intestinal permeability compared to mice treated with antibiotics alone (Fig 7C). When *P*. *aeruginosa* colonized mice were made neutropenic, intestinal permeability significantly increased. This effect was not seen in neutropenic *C*. *albicans* colonized mice. Neutropenic mice co-colonized with *C*. *albicans* and *P*. *aeruginosa* exhibited low intestinal permeability (< 1 ng/uL), and iron supplementation in the co-colonized group significantly increased intestinal permeability (Fig 7C). Finally, neither 1) iron supplementation alone or 2) iron supplementation and *C*. *albicans* colonization in the setting of neutropenia had any significant effect on increasing intestinal permeability. Collectively these results suggest that the iron-induced increase in gut permeability in co-colonized mice was due to *P*. *aeruginosa*. Of note, mice with the highest intestinal permeability (serum-FITC dextran concentrations > 3 ng/uL) were ill-appearing, with signs consistent with acute infection (hunched backs, cold to touch, raised fur). Thus, these findings suggest that iron supplementation restores *P*. *aeruginosa* virulence in *P*. *aeruginosa* and *C*. *albicans* co-colonized mice by modulating gut epithelial integrity (increasing gut permeability) indirectly through *P*. *aeruginosa* and not directly on host intestinal tissue.

## Discussion

Since *P*. *aeruginosa* and *C*. *albicans* co-infect or co-colonize numerous human body niches, we developed a murine model of *P*. *aeruginosa* and *C*. *albicans* GI co-infection that would allow us to better study bacterial-fungal interactions and determine the pathophysiological impact of these interactions. Using this model system, we found that co-colonization with *C*. *albicans* significantly inhibited *P*. *aeruginosa* virulence in the setting of neutropenia—while having no impact on *P*. *aeruginosa* or *C*. *albicans* GI colonization levels. *P*. *aeruginosa* pyoverdine and pyochelin gene expression uniformly decreased when *C*. *albicans* was present in the GI tract. Accordingly, deletion of both pyochelin and pyoverdine genes was sufficient to attenuate *P*. *aeruginosa* virulence. A *C*. *albicans* secreted factor, most likely a secreted protein and not the quorum-sensing molecule farnesol, suppressed *P*. *aeruginosa* pyochelin and pyoverdine gene expression and inhibited pyoverdine production. *C*. *albicans* secreted proteins inhibited *P*. *aeruginosa*-mediated cytotoxic effects (via extracellular effector molecules) against cultured human colonocytes and were sufficient to significantly reduce *P*. *aeruginosa* virulence. Oral iron supplementation restored *P*. *aeruginosa* virulence in *C*. *albicans* and *P*. *aeruginosa* co-colonized mice. Here we describe a novel observation of fungal-inhibition of bacterial effectors critical for virulence but not important for colonization. These findings validate the use of a mammalian model system to investigate complex prokaryotic-eukaryotic interactions in the GI tract.

The antagonistic relationship between *P*. *aeruginosa* and *C*. *albicans* has been well-documented, but largely described with *in vitro* studies. *P*. *aeruginosa* [70,71] or *P*. *aeruginosa* products (notably pyocyanin and 1-hydroxyphenazine) [72] inhibit the growth of *Candida spp*. *in vitro*. Furthermore, *P*. *aeruginosa* forms dense biofilms on *C*. *albicans* hyphae and directly kills the fungus *in vitro* [10]. In fact, both *P*. *aeruginosa* contact-mediated and soluble factors can kill *C*. *albicans* filaments *in vitro* [9]. Interestingly, the yeast form of *C*. *albicans* is resistant to *P*. *aeruginosa* killing [9,10]. In fact, *P*. *aeruginosa* pyocyanin and the quorum-sensing molecule, 3-oxo-C12 homoserine lactone, can promote growth of the yeast form of *C*. *albicans in vitro* [8,72]. In this study, however, co-colonization surprisingly had no effect on *C*. *albicans* or *P*. *aeruginosa’s* individual ability to colonize the GI tract. In terms of rectifying these discrepant findings, the most plausible explanation is that *C*. *albicans* in the murine gut may be predominantly in the yeast form and therefore largely resistant to any *P*. *aeruginosa*-mediated growth inhibition or killing. *C*. *albicans* that colonizes human cutaneous epithelial cell surfaces is predominantly in the yeast form [73,74], and this may also hold true with *C*. *albicans* colonizing GI epithelial surfaces. Future studies that can determine *C*. *albicans* morphology and state (planktonic vs biofilm) in the mammalian GI tract would be immensely informative.

Interestingly, when *P*. *aeruginosa* and *C*. *albicans* form mixed biofilms *in vitro*, *P*. *aeruginosa* pyoverdine, pyochelin, and exotoxin A secretion is significantly increased (thus providing improved iron sequestration); *C*. *albicans* iron acquisition proteins are reduced, and *C*. *albicans* metabolism decreased [75]. Our results differ from these *in vitro* findings for two likely reasons: 1) the microenvironments of *P*. *aeruginosa* and *C*. *albicans* biofilms on plastic versus *P*. *aeruginosa* and *C*. *albicans* in the murine gut are markedly different and may result in significant differences in transcriptional changes and phenotype and 2) *P*. *aeruginosa* and *C*. *albicans* in the murine gut is likely a heterogeneous mix of planktonic, mono biofilm and mixed biofilm microbial populations.

*P*. *aeruginosa* and *C*. *albicans* interactions in *in vivo* models have also been described. For instance, *A*. *baumanii* reduced *C*. *albicans* virulence, and *C*. *albicans* was able to inhibit *A*. *baumanii* growth via farnesol production in a *C*. *elegans* model of co-infection [76]. In contrast, several murine models have demonstrated a synergistic effect of *C*. *albicans*-bacterial co-infection: *C*. *albicans*/*E*. *coli* intravenous bloodstream infection [77,78], *P*. *aeruginosa*/*C*. *albicans* burn wound [79], and *C*. *albicans*/*S*. *aureus* peritonitis [80] models. To the best of our knowledge, our study is the first *in vivo* system to describe a *C*. *albicans*-induced inhibition of *P*. *aeruginosa* iron acquisition proteins resulting in a loss of *P*. *aeruginosa* virulence. Taken together, these prior studies and the data presented here demonstrate the complexity of bacterial-fungal interactions and how significantly they can vary depending on experimental procedures (*in vitro* vs *in vivo)* and body niche/disease model.

A stable gut microbiota promotes pathogenic bacteria colonization resistance, preventing pathogenic microbes from overgrowing and thus decreasing the risk of pathogen virulence. High gut bacterial burden significantly increases the risk of bacteremia (with the same gut bacteria) in immunocompromised patients [29]. *P*. *aeruginosa* is not a normal member of the human commensal microbiota [17,81]. In contrast, the prevalence of *C*. *albicans* GI colonization in humans ranges from less than 10% in remote and traditional societies [82–84] to 40–80% in modern, developed countries [85–87]. Interestingly, adult mice with intact gut microbiota (no antibiotic treatment) are resistant to *P*. *aeruginosa* and *C*. *albicans* [24–26] GI colonization. As with other *P*. *aeruginosa* infections (such as burn wounds, ophthalmic infections, and ventilator associated pneumonia), *P*. *aeruginosa* gut colonization appears to require a deficit in host immune defenses, in this case a disturbance in gut microbial homeostasis. Thus, depletion of anaerobic gut commensals (e.g via antibiotics) is critical for establishing both sustained and high levels *of P*. *aeruginos*a [31] and *C*. *albicans* [24,25] GI colonization in mice.

Perhaps with the depletion of anaerobic commensals (which comprise >99% of the gut microbiota), competition for carbon sources and other nutrients is significantly decreased, creating a more permissive gut environment for colonization. But the question remains: how does *C*. *albicans* benefit from suppressing *P*. *aeruginosa* pyochelin and pyoverdine expression? One possible answer is a competitive advantage for iron. As noted earlier, the host withholds nutrients, such as iron [37], to prevent microbial overgrowth in the gut [38]. Most bacterial pathogens, including *P*. *aeruginosa*, utilize high-affinity iron uptake mechanisms that compete against host-mediated sequestration. *C*. *albicans* possesses similar iron acquisition mechanisms [43,44]. For instance, Als3, a hyphal-associated adhesin and invasin, is also essential in mediating iron acquisition from host ferritin [88]. Thus, in an effort to better secure iron from the host, *C*. *albicans* may secrete a protein that inhibits *P*. *aeruginosa* (and perhaps other microbes) from acquiring iron. Yet, despite inhibition, and even deletion, of pyochelin and pyoverdine genes, *P*. *aeruginosa* is able to grow and colonize the GI tract. As a testament to the importance of iron, *P*. *aeruginosa* employs additional strategies to acquire iron: uptake of xenosiderophores (produced by other organisms)[89–91]; uptake of host heme molecules [92]; FeoABC system (Feo system)[63,93], and extracellular reduction of Fe^3+^ to Fe^2+^ with phenazine compounds [94]. Thus, despite inhibition of pyoverdine and pyochelin by *C*. *albicans*, *P*. *aeruginosa* is probably able to utilize alternative iron acquisition pathways to sustain growth and colonize the GI tract.

While much has been written about the importance of neutropenia [25,31,95,96] and increased bacterial burden [29,62,97] in promoting acute microbial infection, our findings indicate that a rate-limiting step to pathogen virulence can be the ability of the pathogen to breach intact gut mucosal barriers. Intact mucosal barriers are a critical component of innate immunity. Newborn human and mice infants have “leaky” gut epithelium, making them more prone to microbial gut translocation [98–100]. Medications, including cytotoxic chemotherapy, can damage gut epithelium, increasing the risk of bacterial infection. And as noted before, microbial pathogens can produce extracellular molecules that damage host epithelial barriers. In *P*. *aeruginosa*, extracellular proteins exported by the type III secretion system (T3SS) have toxic effects on cultured cells [32,101–103] and can enhance virulence in animal models of *P*. *aeruginosa* pneumonia [104] and gut colonization [30]. We previously demonstrated that disruption of *P*. *aeruginosa* cytotoxic virulence effector genes (e.g. Type III secretion system *ExoU*) results in a loss of cytotoxicity and significantly reduces virulence in this *P*. *aeruginosa* gut model [30], without affecting *P*. *aeruginosa* GI colonization. In the current study, deletion of either pyochelin or pyoverdine decreased *P*. *aeruginosa* cytotoxic activity *in vitro* but virulence was still maintained *in vivo*. However there appears to be an *in vitro* cytotoxic threshold that predicts *in vivo* virulence: wild-type *P*. *aeruginosa* PAO1 (5.8 fold increased cytotoxicity compared to untreated control), PAO1 Δ*pchBA* (5.0), and PAO1 Δ*pvdS* (1.8) all maintained full virulence, whereas wild-type *P*. *aeruginosa* + *C*. *albicans* secreted protein (1.3) virulence was attenuated (Fig 6A).

Hence, we postulate that in *P*. *aeruginosa* and *C*. *albicans* co-colonized mice: *C*. *albicans* secretes one or more factors that inhibit *P*. *aeruginosa* pyochelin and pyoverdine expression. *P*. *aeruginosa* virulence and production of extracellular virulence effectors, such as PrpL and exotoxin A, is decreased, but host gut mucosal integrity remains intact. We further hypothesize that in *P*. *aeruginosa* and *C*. *albicans* co-colonized mice, iron supplementation restores *P*. *aeruginosa* virulence by inducing pyochelin/pyoverdine-independent *P*. *aeruginosa* cytotoxic effector molecules; host gut permeability increases (gut mucosal barriers are damaged); and *P*. *aeruginosa* virulence is restored.

While our data strongly suggests that one or more *Candida albicans* secreted protein(s) mediates the inhibition of *P*. *aeruginosa* virulence, the exact mechanism of pyochelin and pyoverdine gene suppression is unclear. One possible mechanism is microbial-induced modulations in the host oxidative stress response lead to changes in *P*. *aeruginosa* pyochelin and pyoverdine gene expression [105] with reduced production of pyoverdine leading to reduced synthesis of exotoxin A and PrpL protease [55]. Another possibility involves the direct regulation of *P*. *aeruginosa* exotoxin A synthesis. *P*. *aeruginosa* exotoxin A regulator protein, PtxS, inhibits the synthesis of exotoxin A and also autoregulates its synthesis [106,107]. A *C*. *albicans* secreted protein could potentially interfere with the autoregulation of *ptxS* or other regulatory genes involved with exotoxin A synthesis. We have ongoing studies in which we are trying to identify the *C*. *albicans* secreted protein that mediates the effect presented in this study, and we hope to be able to elucidate the specific mechanism in the future.

With advances in medical technology, artificial niches (e.g. the antibiotic-treated gut) are being created where non-commensal microbes (i.e. *P*. *aeruginosa*, *C*. *albicans*) can now establish colonization and in the right setting (e.g. deficits in the immune system) cause invasive disease. Therefore, understanding the synergistic, symbiotic, or antagonistic interactions between diverse microorganisms within clinically relevant environments may be critical for understanding their pathogenesis toward the host. By exploiting specific evolutionary defense mechanisms or survival responses used by competing pathogens, we may gain key insight into novel therapeutic targets for increasingly difficult to treat pathogens such as *P*. *aeruginosa* and *C*. *albicans*.

## Methods

### Bacterial and fungal strains

The *Candida albicans* strains, *Pseudomonas aeruginosa* strains, and other bacteria strains used are listed in S1 Table. Unless otherwise noted, *C*. *albicans* refers to strain SC5314 and *P*. *aeruginosa* refers to strain PAO1. Unless specified otherwise, aerobic bacteria, including *P*. *aeruginosa*, were grown overnight in Luria-Bertani (LB) broth at 37°C; anaerobic bacteria overnight in TYG [108] broth at 37°C anaerobically; and *C*. *albicans* in yeast peptone dextrose (YPD) broth at 30°C.

### Ethics statement

All animal experiments were done in accordance with NIH guidelines, the Animal Welfare Act and US federal law. The University of Texas Southwestern Medical Center’s Institutional Animal Care and Use Committee approved the experimental protocol “2009–0243” that was used for this study. All animals were housed in a centralized and AAALAC-accredited research animal facility that is fully staffed with trained husbandry, technical and veterinary personnel.

### *P*. *aeruginosa* and *C*. *albicans* GI co-colonization model

The *P*. *aeruginosa* and *C*. *albicans* co-colonization and virulence murine model was used as previously described [25,31], with some modifications. Unless otherwise noted, mice used for experiments were C3H/HeN (Harlan), sex-matched, 6–8 weeks of age. Mice within an experiment were littermates that remained co-housed in the same cage to ensure a shared microbiota. Mice were fed sterile water with 2 mg streptomycin /mL (STREP) and 1500 U penicillin G /mL (PCN) for 5 days. *P*. *aeruginosa* and *C*. *albicans* for oral gavage was prepared by harvesting overnight cultures (*P*. *aeruginosa* in LB, 37°C; *C*. *albicans* in YPD, 30°C); washing in PBS x 2; enumerating concentration by spectrophotometry (for *P*. *aeruginosa*) or hemacytometer (*C*. *albicans*); and resuspending the final *P*. *aeruginosa* or *C*. *albicans* oral gavage preparation in endotoxin-free sterile PBS (Gibco). *P*. *aeruginosa* and/or *C*. *albicans* was administered by oral gavage (5 x 10^8^ cfu in 0.2 mL) after 5 days of antibiotic water treatment. Mice were then transitioned to PCN water for the remainder of the experiment.

*C*. *alb*icans and *P*. *aeruginosa* GI colonization were enumerated by first weighing fecal specimens, suspending fecal pellets in 1% proteose peptone, homogenizing samples, serially diluting and culturing homogenized fecal contents on YVG agar (yeast-peptone-dextrose agar with 0.010 mg/mL vancomycin and 0.100 mg/mL gentamicin to suppress bacterial growth) [25] and cetrimide agar, respectively. For experiments utilizing bacteria, obligate anaerobic strains (*B*. *theta*, *B*. *producta*) were cultured in TYG [108] or BHI/Blood agar under anaerobic conditions (Coy anaerobic chamber) at 37°C; *E*. *coli* was grown in LB and MacConkey media under aerobic conditions at 37°C; and *E*. *faecalis* was grown on CNA media under aerobic conditions at 37°C. Bacteria were washed with PBS (aerobic or anaerobic as indicated). Bacterial concentration was determined by spectrophotometry. Bacteria were then administered by oral gavage (5 x 10^8^ cfu). Bacterial colonization levels were enumerated by growth on the appropriate selective media and identity confirmed by gram-stain and enzymatic analysis (Rapid One for Enterobacteriaceae and RapID ANA II for anaerobes, Remel).

For virulence experiments, anti-mouse Ly-6G, Ly-6C (Gr1) monoclonal antibody RB6-8C5 was produced and administered as previously described [25,31]. Mice were monitored for mortality for 7 days. Moribund mice were euthanized with CO_2_ administration. Livers and spleens were resected, homogenized and plated on YVG, MacConkey, TSA, Cetrimide, and BHI/Blood agars. Livers were previously found to be the most reliable organ to confirm *C*. *albicans* extra-intestinal dissemination [25] and spleens the most reliable organ for confirming *P*. *aeruginosa* extra-intestinal dissemination [31] when using this murine model. The presence of a homogeneous population of green, oxidase-positive colonies on cetrimide agar and an absence of other bacterial growth on the MacConkey (aerobic), TSA (aerobic), and BHI/Blood (anaerobic) plates was used for confirmation of *P*. *aeruginosa* dissemination. The presence of a homogeneous population of creamy white colonies on YVG agar was used for evidence of *C*. *albicans* dissemination.

### Intestinal permeability assay

Intestinal barrier function was evaluated by measuring *in vivo* paracellular permeability to fluorescent-labeled dextran as previously described [69]. Mice were fasted for 4.5 hours and then orally gavaged with 50 mg/100g body weight of FITC-dextran (MW 3,000–5000, FD4, Sigma). Serum was obtained 3 hours after gavage administration by terminal cardiac puncture. For neutropenic mice, serum was obtained 84 hours after RB6-8CB monoclonal antibody injection. The serum FITC-dextran concentration was calculated by comparing samples with serial dilutions of known standards using the Synergy HT Fluorimeter (BioTek, Winooski, VT) with excitation at 485 nm and emission at 530 nm.

### *In vivo P*. *aeruginosa* transcriptome profiling

To obtain transcriptome information from *P*. *aeruginosa* colonizing the murine GI tract, we colonized mice with *P*. *aeruginosa* alone or *P*. *aeruginosa* and *C*. *albicans* and then administered RB6-8C5 mAb to induce virulence. When a *P*. *aeruginosa* mouse became moribund, we euthanized this mouse and an accompanying *P*. *aeruginosa* and *C*. *albicans* mouse. Cecal contents were flushed and immediately flash frozen. Cecal contents from 8 mice (1 biological replicate) of the same group were combined, and total RNA was extracted as previously described [109]. Prokaryotic total RNA was further isolated (MICROBEnrich Kit, Ambion). The quality of the resultant RNA was determined using an Agilent Bioanalyzer. For samples with RNA Integrity Numbers of greater than 7.5, the RiboMinus Kit (Life Technologies) was used to deplete rRNA from the total RNA samples. 500 ng of mRNA was used to create cDNA libraries (UTSW Microarray Core). Paired-end libraries for Illumina sequencing were prepared from purified cDNA (TruSeq RNA Sample preparation kit, Illumina). Reads were aligned to *P*. *aeruginosa* PAO1 genome (GenBank: NC_022516.2) using CLC-Biosystems RNA-Seq module. Even after removal of eukaryotic RNA (murine and *C*. *albicans*), <10% of sequence reads properly mapped to the *P*. *aeruginosa* PAO1 genome (S2 Table). The number of overlapping mapped reads was counted for every ORF in each sample of whole-transcriptome sequencing (2 biological replicates for the *P*. *aeruginosa* and *P*. *aeruginosa* and *C*. *albicans* groups). For each ORF the number of reads per kilobase of gene model per million mapped reads (RPKM) was calculated [110]. The raw read counts for each gene was processed using DESeq software (utilizing negative binomial distribution analysis), and differentially expressed genes were identified using adjusted p-values ≤ 0.05 and fold-change ≥ 2 (Benjamini-Hochberg procedure) [111].

### RNA extraction and cDNA synthesis

Total RNA from *P*. *aeruginosa* cells was isolated using phenol-chloroform and bead-beating, followed by ethanol precipitation [112]. Crude RNA extracts were treated with DNaseI (Qiagen) and column purified (RNEasy Kit, Qiagen). In mixed *P*. *aeruginosa* and *C*. *albicans* experiments, prokaryotic total RNA was further isolated (MICROBEnrich Kit, Ambion). RNA concentrations were quantified by spectrophotometry (Nanodrop). Total RNA was used to synthesize cDNA (iScript, BioRad).

### RT-qPCR

Total RNA was used to synthesize cDNA (iScript, BioRad). qPCR analysis was performed using the SsoAdvanced SYBR Green Supermix (Bio-Rad) and specific primers (S3 Table). Signals were normalized to *P*. *aeruginosa rpoD* [113] levels within each sample and normalized data were used to calculate relative levels of gene expression using ΔΔC_t_ analysis.

### *Pseudomonas aeruginosa gyrB* qPCR

The abundance of the *P*. *aeruginosa gyrB* gene was determined by qPCR analysis (SsoAdvanced Supermix, Bio-Rad) using *P*. *aeruginosa gyrB* specific gene primers and specific probe (S3 Table). To confirm specificity, *P*. *aeruginosa gyrB* qPCR was performed using gDNA from *Escherichia coli* ATC10798, *Enterobacter cloacae* (clinical isolate), *Enterococcus faecalis* (clinical isolate), and *Candida albicans* SC5314, and no false positives were noted (Fig 4B). *P*. *aeruginosa* abundance was determined using standard curves constructed with reference to cloned DNA corresponding to a short segment of *P*. *aeruginosa gyrB* gene (S3 Table). Note that qPCR measures gene copies/g tissue, not actual bacterial/fungal numbers or colony forming units.

To test *P*. *aeruginosa gyrB* levels in murine feces and intestinal tissue, fecal specimens and intestinal tract segments (2 cm; ileum, cecum, proximal and distal colon) were collected. Intestinal segments were cut open longitudinally and flushed with ice-cold sterile PBS x 2. Tissue was then flash frozen with liquid nitrogen, weighed, and immediately suspended in extraction buffer (200 mM NaCl, 200mM Tris, 20 mM EDTA, 6% SDS) and 0.5 ml of phenol/chloroform/isoamyl alcohol, pH 7.9 (Ambion) [108]. Tissue was lysed by bead-beating (0.1 mm zirconia/silica beads, BioSpec), and subjected to additional phenol/chloroform extractions. Crude DNA extracts were treated with RNAseA (Qiagen) and column-purified (PCR Purification Kit, Qiagen). DNA concentrations were quantified by fluorescence-based assay (Quant-iT PicoGreen dsDNA, Life Technologies).

### *In vitro P*. *aeruginosa* and *C*. *albicans* co-culture *P*. *aeruginosa* gene expression experiments

*P*. *aeruginosa* and *C*. *albicans* were individually grown to mid-log phase. *P*. *aeruginos*a was grown in GGP media (3% glycerol, 1% proteose peptone, 2.9 mM K_2_HPO_4_, 2.0 mM MgSO_4_⚫7H_2_0) at 37°C. *C*. *albicans* was grown in YPD media at 30°C. *P*. *aeruginosa* and *C*. *albicans* cultures were combined in a 1:1 ratio. The *P*. *aeruginosa*/*C*. *albicans* culture was grown at 37°C, and cells harvested after 10 minutes of co-incubation. Total RNA was extracted, and prokaryotic RNA was isolated (MICROBEnrich Kit, Ambion) for RT qPCR of *P*. *aeruginosa* pyochelin and pyoverdine gene expression.

### *P*. *aeruginosa* pyoverdine and/or pyochelin mutant construction

*P*. *aeruginosa* mutant strains and plasmids used are listed in S4 Table. Methods for construction of unmarked deletions in *pchBA* and *pvdH* have been described previously[114,115]. Fragments of DNA flanking each deletion site were amplified by PCR, ligated together, cloned into the allele replacement vectors pEX18Gm [114] and used to replace the wild-type genes in *P*. *aeruginosa* PAO1.

To generate PAO6901 and PAO6902, the Gm^r^ cassette from PAO1ΔpvdS::Gm^r^ [92] was transduced via E79tv-2 [116] into PAO1 and PAO6297, respectively.

### Complementation of *P*. *aeruginosa* mutants

To generate plasmid pME6477 for complementation experiments, the pchDCBA operon was fused at the ATG start codon of *pchD* to the lac promoter (Plac) of pME6000 by using a linker region containing a ribosome binding site (AAGCTTGATATCGAATTGTGAGCGGATAACAATTTCACACAGAATTGATTAAAGAGGAGAAATTAAGCATG; the HindIII site used for cloning into pME6000 is italicized; the ATG start codon of *pchD* is underlined).

For complementation of pyochelin/pyoverdine double knockout mutants, PAO1 cells were grown in LB broth to stationary phase, harvested, washed 0.1 M MgCl_2_, and finally suspended in ice-cold transformation buffer (75 mM CaCl_2_, 6mM MgCl_2_, 15% glycerol) [117]. 100 μL of the PAO1 competent cells were then transferred to thin-walled 13 x 100 mm borosilicate glass tubes that were pre-chilled on ice. 2–5 μL aliquots containing 100–1000 ng pME6477 (pME6000 derivative carrying *pchDCBA*) that had been purified using the PureYield Plasmid Midiprep kit (Promega) was then added. DNA-cell mixture was incubated on ice for 15 min and then heat-shocked at 37°C for 2 min. 500 μL LB broth was immediately added, and tubes were incubated at 37°C for 1 h in a shaking incubator. After incubation, a 200-μ L aliquot of each undiluted and diluted (1:10) suspension was plated on LB agar containing 100 μg/mL tetracycline. Plates were incubated at 37°C for 24 h. Select colonies were seeded into LB/Tetracycline media and grown at 37°C. Plasmids were isolated (PureYield Plasmid Miniprep Kit, Promega). The identity of plasmids were confirmed by both gel electrophoresis and sequencing.

### Quantitative iron assay

Fecal iron content was measured using a colorimetric ferrozine-based assay [118,119]. Mouse fecal pellets, mouse chow (Teklad Global 16% Protein Rodent Diet, Harlan; 1 gram), liquid media (1 ml), and antibiotic water (1 ml) were collected, weighed, homogenized in 6M HCl, and stored under anaerobic conditions for 24 hours. 0.1 ml of filtered fecal homogenate was then added to 0.9 ml of hydroxylamine hydrochloride (HAHC) (10%, w/v, in 1M HCl). 1 ml of ferrozine solution was added and absorbance at 562nm was measured. Total Fe was calculated in reference to standard curves and reported as mmol/mg feces

### Iron depletion

YPD and *C*. *albicans* supernatant proteins were passed over a column with Chelex100 50–100 mesh[69] (Sigma) resin beds as per the manufacturer’s instructions. Iron concentrations were measured using the ferrozine assay described above.

### Pyoverdine assay

*P*. *aeruginosa* cells were grown in GGP medium[120], in which limited iron availability enhances the expression of pyochelin and pyoverdine, in black, clear bottom 96-well plates (Corning Incorporated, Costar 3603). Pyoverdine was measured by fluorescence at 400±10/460±40 nm excitation/emission, using a 96-well Microplate Fluorimeter Plate Reader (Synergy HT, Biotek Inc.), and measurements of relative fluorescence units (RFU) were normalized to cell density measured at 600 nm. Measurements were recorded dynamically up to 24 h. Between measurements, plates were incubated at 37°C and 100 rpm. The specificity of fluorescence for pyoverdine was verified using PAO1*ΔpvdS* mutant deficient in pyoverdin production, in which no fluorescence was found.

### Heat-killed *C*. *albicans*

For heat inactivation and killing, *C*. *albicans* (yeast or hyphae) were resuspended in phosphate buffered saline (PBS) (1 x 10^7^ cells/ml) and treated at 100°C for 1 h [45]. Killing was confirmed by plating the heat-killed on *C*. *albicans* on YPD agar and confirming lack of growth.

### Microbial extracellular protein isolation

Microbial extracellular proteins were isolated as previously described [31]. *P*. *aeruginosa* strains, *C*. *albicans* (SC5314), and *E*. *coli* (ATCC 10798) were grown to stationary phase in the following media and conditions: GGP at 37°C for P. aeruginosa; YPD at 30°C for *C*. *albicans*; and LB at 37°C for *E*. *coli*. Supernatants were prepared from the cultures by centrifugation at 10,000 x *g* at 4°C for 30 min. *C*. *albicans* culture supernatant was then sterile filtered (0.22 μm, low-protein binding Steritop filter; Millipore). Proteins present in the supernatants were precipitated by the addition of ammonium sulfate (50% for bacterial supernatants, 80% for *C*. *albicans* supernatant) and left overnight at 4°C. Precipitated material was isolated by centrifugation at 10,000 x *g* at 4°C for 30 min. Protein pellets were resuspended in distilled H2O and run through PD-10 desalting columns containing Sephadex G-25 gel (Amersham Biosciences). Protein preparations then underwent two rounds of dialysis in PBS buffer (Slide-A-Lyzer Dialysis cassette, 10K MWCO; Thermo Scientific). Protein concentrations were determined by BCA protein assay (Pierce).

### Physical and chemical denaturation of *C*. *albicans* supernatant proteins

For physical denaturation, *C*. *albicans* supernatant protein preparations were boiled for 60 minutes. For chemical denaturation, *C*. *albicans* supernatant protein preparations were treated with protease from *Streptomyces griseus* (a mixture of at least three proteolytic activities including an extracellular serine protease; Sigma P5147) at a final concentration of 5mg/mL for 60 minutes at 37°C, then heat-inactivated at 80°C for 15 minutes. Equivalent volumes (the same volume of the corresponding untreated C. albicans supernatant protein) of boiled or protease-treated *C*. *albicans* supernatant proteins were added to *P*. *aeruginosa* cultures.

### *In vitro P*. *aeruginosa* and *C*. *albicans* supernatant/supernatant protein *P*. *aeruginosa* gene expression experiments

For experiments utilizing live *P*. *aeruginosa* and *C*. *albicans supernatant* (Fig 5D), *C*. *albicans* culture supernatant was prepared from an overnight *C*. *albicans* culture (grown in YPD, 30°C) by centrifugation at 10,000 x *g* at 4°C for 30 min. *C*. *albicans* culture supernatant was then sterile filtered (0.22 μm, low-protein binding Steritop filter; Millipore) and then dialyzed against PBS (Slide-A-Lyzer Dialysis cassette, 10K MWCO; Thermo Scientific). *P*. *aeruginosa* was grown to mid-log phase in GGP media at 37°C. *P*. *aeruginosa* and *C*. *albicans* supernatant was combined in a 1:1 ratio. The P. *aeruginosa/C*. *albican*s supernatant culture was grown at 37°C, and cells harvested after 10 minutes of co-incubation for RNA extraction (as described above).

For experiments utilizing live *P*. *aeruginosa* and *C*. *alb*icans supernatant protein (Fig 5D and 5E), *C*. *albicans* culture supernatant proteins were prepared as described above. *P*. *aeruginosa* was grown to mid-log phase in GGP media at 37°C. *C*. *albicans* supernatant protein was added to a final concentration of 100 ug/mL. The *P*. *aeruginosa* and *C*. *albicans* supernatant protein culture was grown at 37°C, and cells harvested after 10 minutes of co-incubation for RNA extraction (as described above).

### Exotoxin A immunoblot

For detection of *P*. *aeruginosa* exotoxin A protein, nitrocellulose membranes were activated by soaking in TBS-0.05% Tween. A slot blotter was used to load samples of 250 ug of total extracellular protein material. The membrane was blocked with TBS containing 5% BSA at room temperature for 1 h. The primary antibody used was a polyclonal rabbit IgG to *P*. *aeruginosa* exotoxin A (Sigma, P2318) diluted 1:10,000, and the blot was rotated at room temperature for 90 min. The secondary antibody was an anti-rabbit whole-molecule IgG conjugated to alkaline phosphatase diluted 1:50,000, which was left on the blot for 90 min at 37°C. After washing, a phosphatase substratedeveloper (KPL) was then added. Relative density of exotoxin A immunoblot was measured by ImageJ Software.

### *In vitro* cytotoxicity assays

Human colonocyte cell lines, HT-29, were exposed to microbial culture supernatant proteins (1 μg/uL) and incubated for 3 hours at 37°C with 5% CO_2_. Cell toxicity was measured by CytoTox-Glo assay (Promega).

### Accession numbers

Accession numbers for the genes described in this study in NCBI are *pchD* (880566), *fptA* (880419), *ampP* PA4218 (880532), *pchF* (88083), *pchH* (880074), *pchA* (881821), *pchE* (880048), *pchG* (880082), *pchB* (881846), *pchC* (881845), *pvdL* (882838), *pvdH* (882857), *pvdS* (882839), *fpvA* (878605), *pvdD* (879750), *pvdF* (878624), *pvdE* (877645), *gbuA* (881747), PA4280.1 (3240240), PA1412 (879323), PA1387 (879767), *hcpB* (879579), PA1316 (881564), PA4722 (881593), *hsiA2* (882166), *aprA* (881248), PA2693 (882826), *hsiC2* (879592), *hcp1* (879446), *oprH* (878005), PA3190 (882901), *pqsH* (879540), *hsiB2* (879559), *clpV1* (879553), *tssB1* (879476), *cbpD* (877771), PA2463 (882955), *pvdP* (882224), *pvdA* (882167), *fumC1* (881033), *lasI* 881777), *fpvA* (878605), PA0572 (878237), PA2402 (878257), Exotoxin A (877850), prpL (880208)

Raw RNA-Seq data is deposited at the NCBI Sequence Read Archive (SRA): SRP061622.

## Supporting Information

S1 Fig Commensal bacteria inhibit *P*. *aeruginosa* Colonization in the Murine GI Tract. *P*. *aeruginosa* PAO1 (red circles) and commensal bacteria (black squares) GI colonization levels in adult antibiotic-treated mice (C3H/HeN). Mice initially colonized with *P*. *aeruginosa* were gavaged with commensal bacteria (5 x 10^8^ cfu) and then transitioned to sterile water. Microbial GI colonization levels were checked 7 day later. n = 8 mice per group. Points represent results from individual animals. Horizontal lines with bars represent the median with interquartile range. Statistical analysis performed by Mann-Whitney test. * p< 0.05; ** p<0.01; ns, not significant. PA = *P*. *aeruginosa*, EC = *Escherichia coli* ATCC 10798D, EF = *Enterococcus faecalis* clinical isolate, BT = *Bacteroides thetaiotamicron* VPI-5482, BP = *Blautia producta* ATCC 27340D.
(TIF)

S2 Fig Microbial GI colonization levels in *P*. *aeruginosa* and *C*. *albicans* co-colonized mice. *C*. *albicans* (red triangles) and *P*. *aeruginosa* (black circles) GI colonization levels in C3H/HeN mice treated with antibiotics and colonized with *P*. *aeruginosa* and/or *C*. *albicans*. **A**) *P*. *aeruginosa* PAO1, **B**) *P*. *aeruginosa* PA14, and **C**) *P*. *aeruginosa* PAK ± *C*. *albicans* strains Can091, Can098, or 3153A. n = 8 mice per group. Points represent results from individual animals. Horizontal lines with bars represent the median with interquartile range. Statistical analysis performed by Mann-Whitney test. * p< 0.05; ** p<0.01; ns, not significant.
(TIF)

S3 Fig Survival curves of neutropenic *P*. *aeruginosa* and *C*. *albicans* co-colonized mice, and *P*. *aeruginosa* levels in spleens of deceased *P*. *aeruginosa* and *C*. *albicans* co-colonized mice. C3H/HeN mice were treated with oral antibiotics and then co-colonized with *P*. *aeruginosa* and *C*. *albicans*. (**A**, **C**, **E**) Survival curves of neutropenic C3H/HeN mice co-colonized with *P*. *aeruginosa* and *C*. *albicans*. **A**) *P*. *aeruginosa* PAO1, **C**) *P*. *aeruginosa* PA14, and **E**) *P*. *aeruginosa* PAK ± *C*. *albicans* strains Can091, Can098, or 3153A. n = 8 mice per group. Survival curves analyzed by log-rank test. * p< 0.05; ** p<0.01; ns, not significant. **B**, **D**, **F**) *P*. *aeruginosa* levels in spleens of deceased neutropenic antibiotic-treated mice colonized with *P*. *aeruginosa* ± *C*. *albicans*. B) PAO1, D) PA14, and F) PAK ± Can091, Can098, or 3153A. The presence of a homogeneous population of green, oxidase-positive colonies on cetrimide agar and an absence of other bacterial growth on the MacConkey (aerobic), TSA (aerobic), and BHI/Blood (anaerobic) plates was used for confirmation of *P*. *aeruginosa* dissemination. Points represent results from individual animals. n = 8 mice per group. Horizontal lines with bars represent the median with interquartile range.
(TIF)

S4 Fig The effect of *C*. *albicans* on *E*. *coli* GI colonization and virulence in mice. **A**) *C*. *albicans* SC5314 (red triangles) and *Escherichia coli*, clinical isolate recovered from the bloodstream of a pediatric cancer patient (black circles), GI colonization levels in antibiotic-treated mice (C3H/HeN). n = 8 mice per group. Points represent results from individual animals. Horizontal lines with bars represent the median with interquartile range. Statistical analysis performed by Mann-Whitney test. * p< 0.05; ** p<0.01; ns, not significant. CA, *C*. *albicans*. **B**) Survival curves of neutropenic C3H/HeN mice colonized with *E*. *col*i ± *C*. *albicans* SC5314. n = 8 mice per group. Statistical analysis performed by log-rank test. * p< 0.05; ** p<0.01; ns, not significant.
(TIF)

S5 Fig *C*. *albicans* quorum-sensing molecule farnesol does not modulate *P*. *aeruginosa* GI colonization or virulence. **A)** *P*. *aeruginosa* PAO1 (circles) and **B)**. *C*. *albicans* SC5314 (red triangles) GI colonization levels in mice (C3H/HeN) co-colonized with PAO1 and *C*. *albicans* SN152, PAO1 and *C*. *albicans dpp3* ^Δ/Δ^ (KWN2), or PAO1 and *C*. *albicans dpp3* ^Δ/Δ^ complemented (KWN4) strains. n = 8 mice per group. PA, *P*. *aeruginosa*. CA, *C*. *albicans*. **C**. Survival curves of neutropenic mice (C3H/HeN) co-colonized with *P*. *aeruginosa* ± *C*. *albicans* SN152, *C*. *albicans dpp3* ^Δ/Δ^ (KWN2), or PAO1 and *C*. *albicans dpp3* ^Δ/Δ^ (KWN4) n = 8 mice per group. **D**. *P*. *aeruginosa* PAO1 (circles) GI colonization levels in mice (C3H/HeN) treated ± farnesol for 7 days [47]. n = 8 mice per group. **E**. Survival curves of neutropenic mice (C3H/HeN) colonized with *P*. *aeruginosa* PAO1 ± farnesol for 7 days [47]. n = 8 mice per group. For GI colonization data, points represent results from individual animals. Horizontal lines with bars represent the median with interquartile range. Statistical analysis performed by Mann-Whitney test. For survival curve data, statistical analysis performed by log-rank test. * p< 0.05; ** p<0.01; ns, not significant.
(TIF)

S6 Fig Complementation with pyochelin genes *pchBA* in pyochelin/pyoverdine deletional mutants restores *P*. *aeruginosa* virulence. **A, B, C, E) (A)** *P*. *aeruginosa* PAO1 pyochelin mutant, (**B**) pyoverdine mutant, (**C**) pyochelin/pyoverdine mutant, and **(E)** pyochelin/pyoverdine knockout complemented strain levels in spleens of deceased neutropenic antibiotic-treated mice colonized with respective *P*. *aeruginosa* mutant strains. The presence of a homogeneous population of green, oxidase-positive colonies on cetrimide agar and an absence of other bacterial growth on the MacConkey (aerobic), TSA (aerobic), and BHI/Blood (anaerobic) plates was used for confirmation of *P*. *aeruginosa* dissemination. n = 8 mice per group. Points represent results from individual animals. Horizontal lines with bars represent the median with interquartile range. **(D)** Survival curves of neutropenic C3H/HeN mice GI colonized with PAO1 Δ*pchBA*Δ*pvdF* pME6477 (*pchBA*), PAO1 Δ*pchBA*Δ*pvdS* pME6477 (*pchBA*), and PAO1 Δ*pchBA*Δ*pvdH* pME6477 (*pchBA*), and WT PAO1. n = 8 mice per group. Statistical analysis performed by log-rank test. ns, not significant.
(TIF)

S7 Fig Hyphal *C*. *albicans* inhibits *P*. *aeruginosa* pyochelin and pyoverdine gene expression *in vitro*. Pyochelin and pyoverdine gene expression by RT qPCR of P. *aeruginosa* PAO1 grown *in vitro* in GGP media to mid-log phase ± *C*. *albicans* (yeast), *C*. *albicans* (hyphal). Yeast *C*. *albicans* grown in YPD at 30°C. Hyphal *C*. *albicans* grown in YPD/10% fetal calf serum (FCS) at 37°C. *C*. *albicans* added to *P*. *aeruginosa* culture in 1:1 ratio and co-incubated at 37°C for 10 minutes before RNA extraction. Bars are means ± SEM. Assays were performed in triplicate. Statistical analysis was performed by unpaired Student’s *t-test*. * p< 0.05; ** p<0.01; ns, not significant.
(TIF)

S8 Fig Iron in YPD media inhibits *P*. *aeruginosa* pyochelin and pyoverdine gene expression. **A)** Total iron content of YPD media, YPD media depleted of iron (Chelex100, Sigma), and iron-depleted YPD media supplemented with iron. Total iron content (Fe^2+^and Fe^3+^) was determined by ferrozine assay. **B)** Pyochelin and pyoverdine gene expression by RT qPCR of *P*. *aeruginosa* PAO1 grown *in vitro* to mid-log phase in iron-limited GGP media with or without YPD media, YPD media depleted of iron (Chelex100, Sigma), and iron-depleted YPD media supplemented with iron. All data shown are means + SEM. Assays were performed in triplicate. Statistical analysis was performed by unpaired Student’s *t-test*. * p< 0.05; ** p<0.01; ns, not significant.
(TIF)

S9 Fig *C*. *albicans* supernatant proteins do not inhibit *P*. *aeruginosa* growth *in vitro*. *P*. *aeruginosa* PAO1 grown *in vitro* in GGP media ± *C*. *albicans* supernatant proteins (0, 1, 10, and 100 μg/mL) at 37°C, 200 rpm. OD_600_ were measured at the time points shown. All data shown are means ± SEM. Assays were performed in triplicate.
(TIF)

S10 Fig Physical and chemical denaturation of *C*. *albicans* supernatant proteins abrogates the *C*. *albicans* supernatant protein induced inhibition of *P*. *aeruginosa* pyochelin and pyoverdine gene expression. Relative pyoverdine production (as determined by measuring fluorescence at 400±10/460±40 nm excitation/emission and normalizing to cell density measured at 600 nm) of *P*. *aeruginosa* grown in GGP media at 37°C over 24 hours with *C*. *albicans* supernatant protein (SP, final concentration 100 ug/mL), boiled *C*. *albicans* supernatant protein (SP/B, boiled for 60 minutes), or *C*. *albicans* supernatant protein treated with *Streptomyces griseus* protease (SP/P, for 60 minutes) compared to an untreated *P*. *aeruginosa* control. A) *P*. *aeruginosa* PAO1, B) *P*. *aeruginosa* PA14, and C) *P*. *aeruginosa* PAK ± *C*. *albicans* supernatant proteins from *C*. *albicans* strains Can091, Can098, or 3153A. All data shown are means ± SEM. Assays were performed in triplicate. Statistical analysis was performed by unpaired Student’s t-test. * p< 0.05; ** p<0.01; ns, not significant.
(TIF)

S1 Table Bacterial and fungal strains used in this study. (PDF)

S2 Table RNA-Seq mapping statistics. (PDF)

S3 Table Primers used in this study. (PDF)

S4 Table *Pseudomonas aeruginosa* strains and plasmids used in this study to generate PA pyochelin/pyoverdine knockout mutants. (PDF)

S1 File *In vivo* RNA-Seq results. (XLSX)
