# Supplementary figures and images for: Candida albicans Inhibits Pseudomonas aeruginosa Virulence through Suppression of Pyochelin and Pyoverdine Biosynthesis
Source: PLoS Pathog. 2015 Aug 27;11(8):e1005129. doi: 10.1371/journal.ppat.1005129 (PMC4552174; doi:10.1371/journal.ppat.1005129)

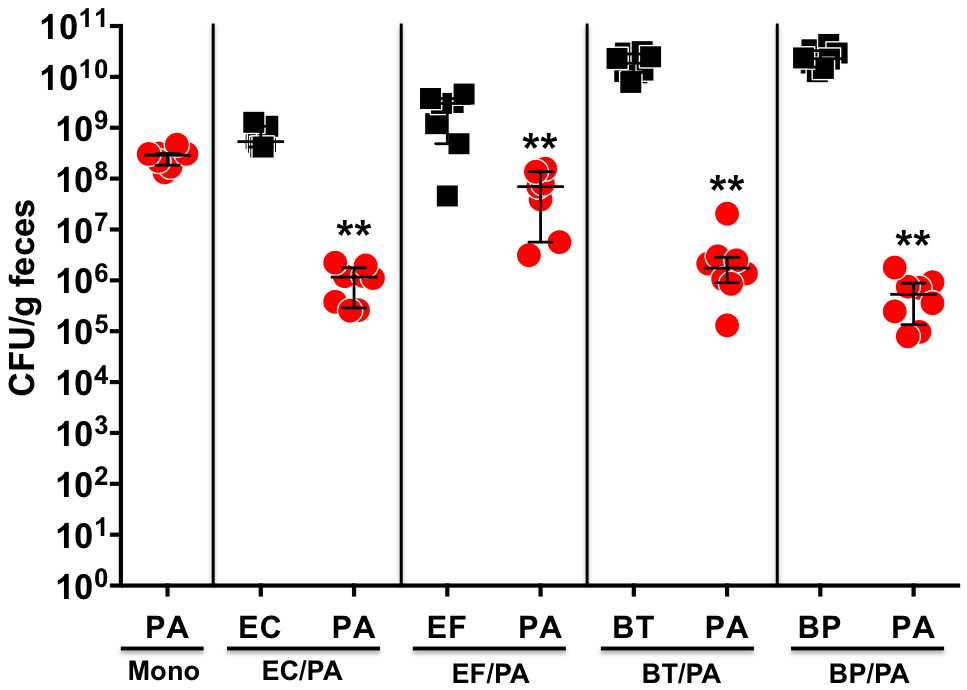

Supplement: S1 Fig — P. aeruginosa PAO1 (red circles) and commensal bacteria (black squares) GI colonization levels in adult antibiotic-treated mice (C3H/HeN). Mice initially colonized with P. aeruginosa were gavaged with commensal bacteria (5 x 10^8^ cfu) and then transitioned to sterile water. Microbial GI colonization levels were checked 7 day later. n = 8 mice per group. Points represent results from individual animals. Horizontal lines with bars represent the median with interquartile range. Statistical analysis performed by Mann-Whitney test. * p< 0.05; ** p<0.01; ns, not significant. PA = P. aeruginosa, EC = Escherichia coli ATCC 10798D, EF = Enterococcus faecalis clinical isolate, BT = Bacteroides thetaiotamicron VPI-5482, BP = Blautia producta ATCC 27340D. (TIF) [file ppat.1005129.s001.tif]

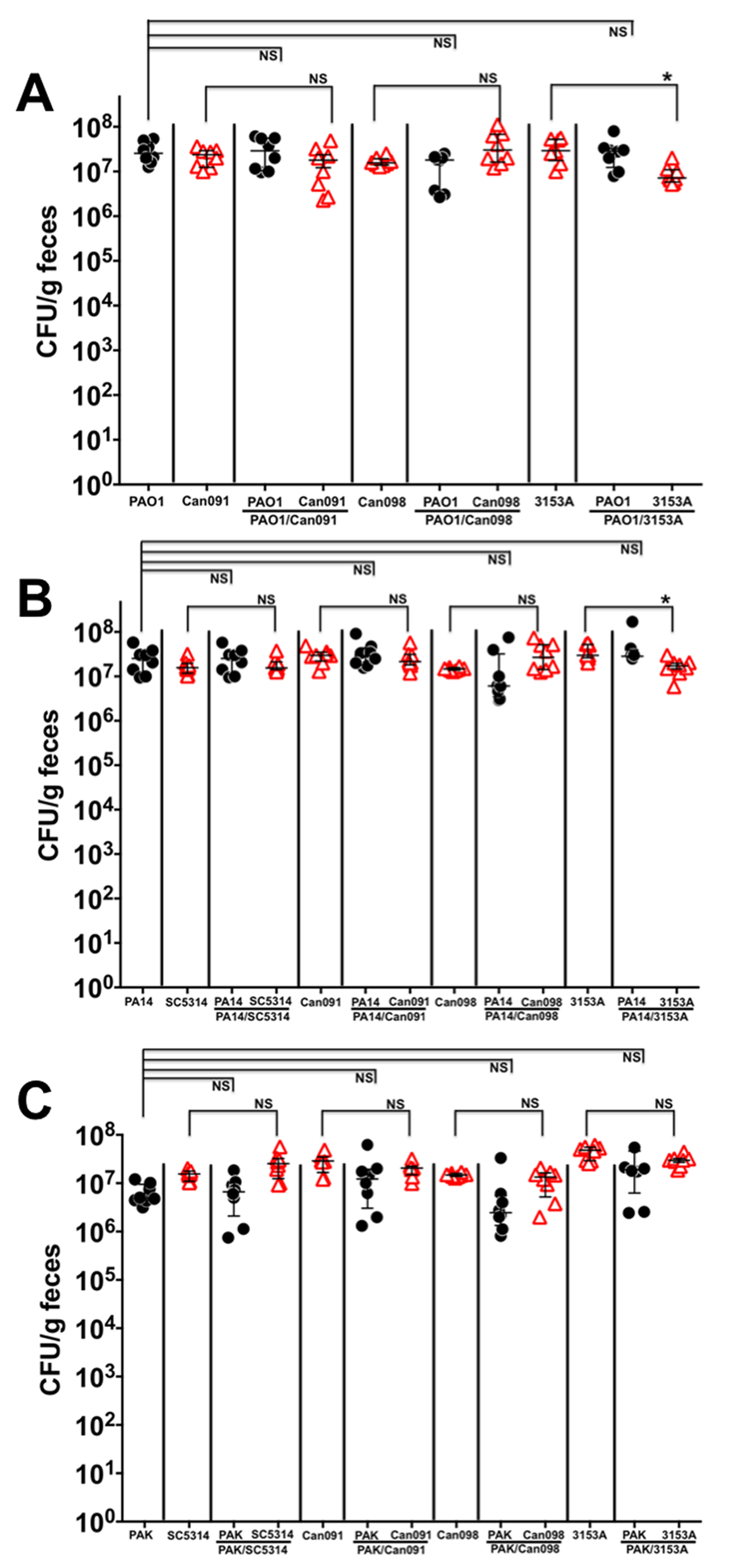

Supplement: S2 Fig — C. albicans (red triangles) and P. aeruginosa (black circles) GI colonization levels in C3H/HeN mice treated with antibiotics and colonized with P. aeruginosa and/or C. albicans. A) P. aeruginosa PAO1, B) P. aeruginosa PA14, and C) P. aeruginosa PAK ± C. albicans strains Can091, Can098, or 3153A. n = 8 mice per group. Points represent results from individual animals. Horizontal lines with bars represent the median with interquartile range. Statistical analysis performed by Mann-Whitney test. * p< 0.05; ** p<0.01; ns, not significant. (TIF) [file ppat.1005129.s002.tif]

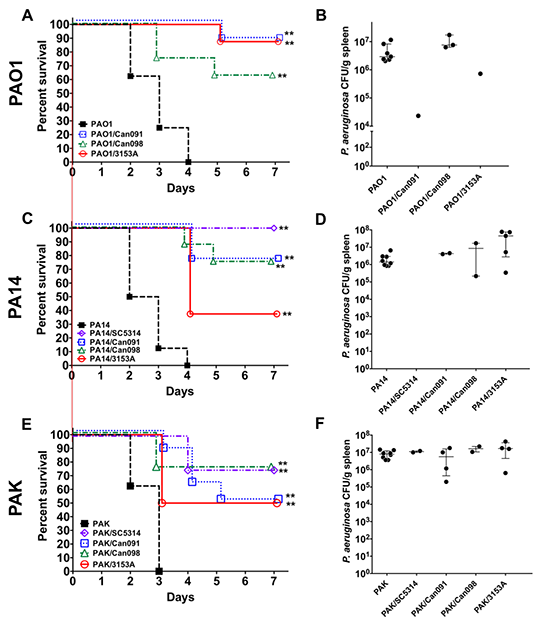

Supplement: S3 Fig — C3H/HeN mice were treated with oral antibiotics and then co-colonized with P. aeruginosa and C. albicans. (A, C, E) Survival curves of neutropenic C3H/HeN mice co-colonized with P. aeruginosa and C. albicans. A) P. aeruginosa PAO1, C) P. aeruginosa PA14, and E) P. aeruginosa PAK ± C. albicans strains Can091, Can098, or 3153A. n = 8 mice per group. Survival curves analyzed by log-rank test. * p< 0.05; ** p<0.01; ns, not significant. B, D, F) P. aeruginosa levels in spleens of deceased neutropenic antibiotic-treated mice colonized with P. aeruginosa ± C. albicans. B) PAO1, D) PA14, and F) PAK ± Can091, Can098, or 3153A. The presence of a homogeneous population of green, oxidase-positive colonies on cetrimide agar and an absence of other bacterial growth on the MacConkey (aerobic), TSA (aerobic), and BHI/Blood (anaerobic) plates was used for confirmation of P. aeruginosa dissemination. Points represent results from individual animals. n = 8 mice per group. Horizontal lines with bars represent the median with interquartile range. (TIF) [file ppat.1005129.s003.tif]

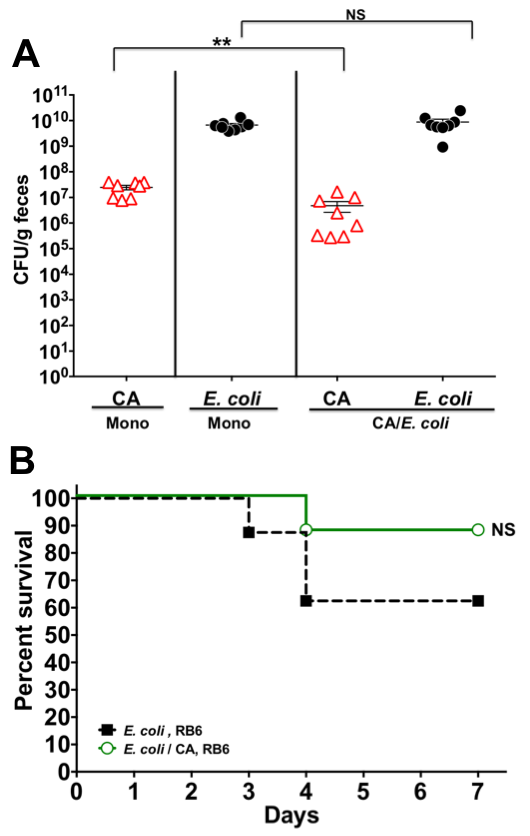

Supplement: S4 Fig — A) C. albicans SC5314 (red triangles) and Escherichia coli, clinical isolate recovered from the bloodstream of a pediatric cancer patient (black circles), GI colonization levels in antibiotic-treated mice (C3H/HeN). n = 8 mice per group. Points represent results from individual animals. Horizontal lines with bars represent the median with interquartile range. Statistical analysis performed by Mann-Whitney test. * p< 0.05; ** p<0.01; ns, not significant. CA, C. albicans. B) Survival curves of neutropenic C3H/HeN mice colonized with E. coli ± C. albicans SC5314. n = 8 mice per group. Statistical analysis performed by log-rank test. * p< 0.05; ** p<0.01; ns, not significant. (TIF) [file ppat.1005129.s004.tif]

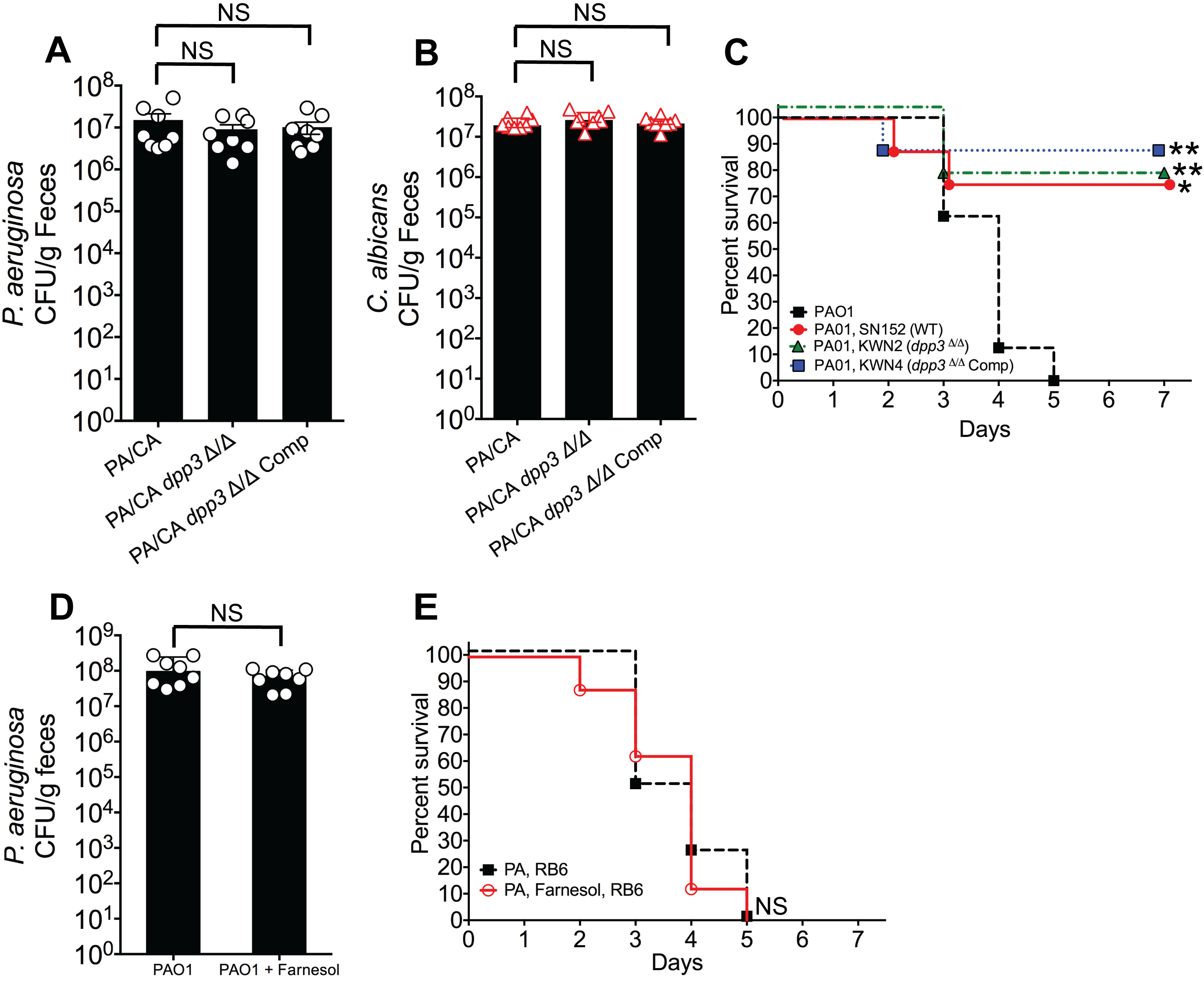

Supplement: S5 Fig — A) P. aeruginosa PAO1 (circles) and B). C. albicans SC5314 (red triangles) GI colonization levels in mice (C3H/HeN) co-colonized with PAO1 and C. albicans SN152, PAO1 and C. albicans dpp3 Δ/Δ (KWN2), or PAO1 and C. albicans dpp3 Δ/Δ complemented (KWN4) strains. n = 8 mice per group. PA, P. aeruginosa. CA, C. albicans. C. Survival curves of neutropenic mice (C3H/HeN) co-colonized with P. aeruginosa ± C. albicans SN152, C. albicans dpp3 Δ/Δ (KWN2), or PAO1 and C. albicans dpp3 Δ/Δ (KWN4) n = 8 mice per group. D. P. aeruginosa PAO1 (circles) GI colonization levels in mice (C3H/HeN) treated ± farnesol for 7 days [47]. n = 8 mice per group. E. Survival curves of neutropenic mice (C3H/HeN) colonized with P. aeruginosa PAO1 ± farnesol for 7 days [47]. n = 8 mice per group. For GI colonization data, points represent results from individual animals. Horizontal lines with bars represent the median with interquartile range. Statistical analysis performed by Mann-Whitney test. For survival curve data, statistical analysis performed by log-rank test. * p< 0.05; ** p<0.01; ns, not significant. (TIF) [file ppat.1005129.s005.tif]

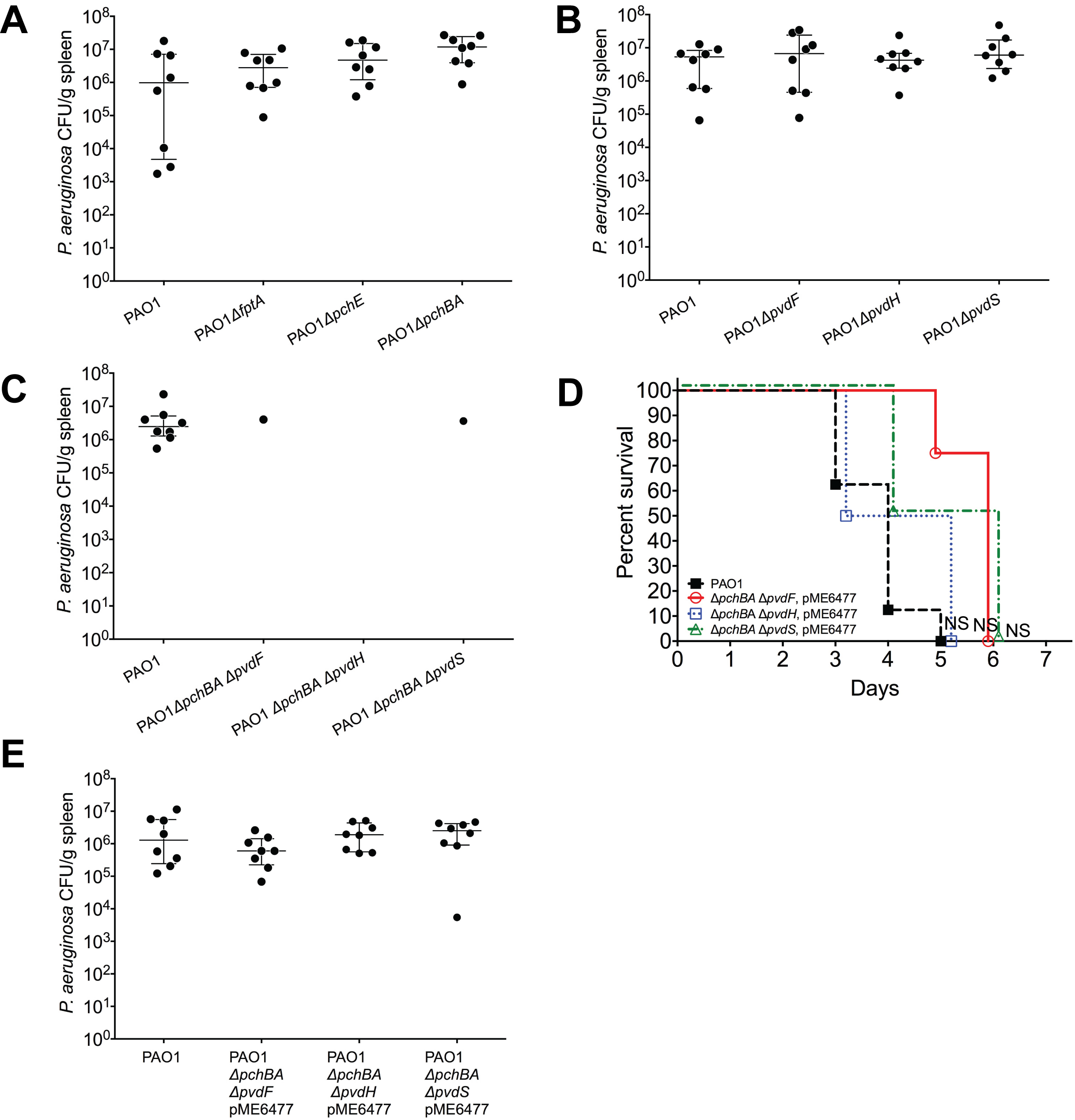

Supplement: S6 Fig — A, B, C, E) (A) P. aeruginosa PAO1 pyochelin mutant, (B) pyoverdine mutant, (C) pyochelin/pyoverdine mutant, and (E) pyochelin/pyoverdine knockout complemented strain levels in spleens of deceased neutropenic antibiotic-treated mice colonized with respective P. aeruginosa mutant strains. The presence of a homogeneous population of green, oxidase-positive colonies on cetrimide agar and an absence of other bacterial growth on the MacConkey (aerobic), TSA (aerobic), and BHI/Blood (anaerobic) plates was used for confirmation of P. aeruginosa dissemination. n = 8 mice per group. Points represent results from individual animals. Horizontal lines with bars represent the median with interquartile range. (D) Survival curves of neutropenic C3H/HeN mice GI colonized with PAO1 ΔpchBAΔpvdF pME6477 (pchBA), PAO1 ΔpchBAΔpvdS pME6477 (pchBA), and PAO1 ΔpchBAΔpvdH pME6477 (pchBA), and WT PAO1. n = 8 mice per group. Statistical analysis performed by log-rank test. ns, not significant. (TIF) [file ppat.1005129.s006.tif]

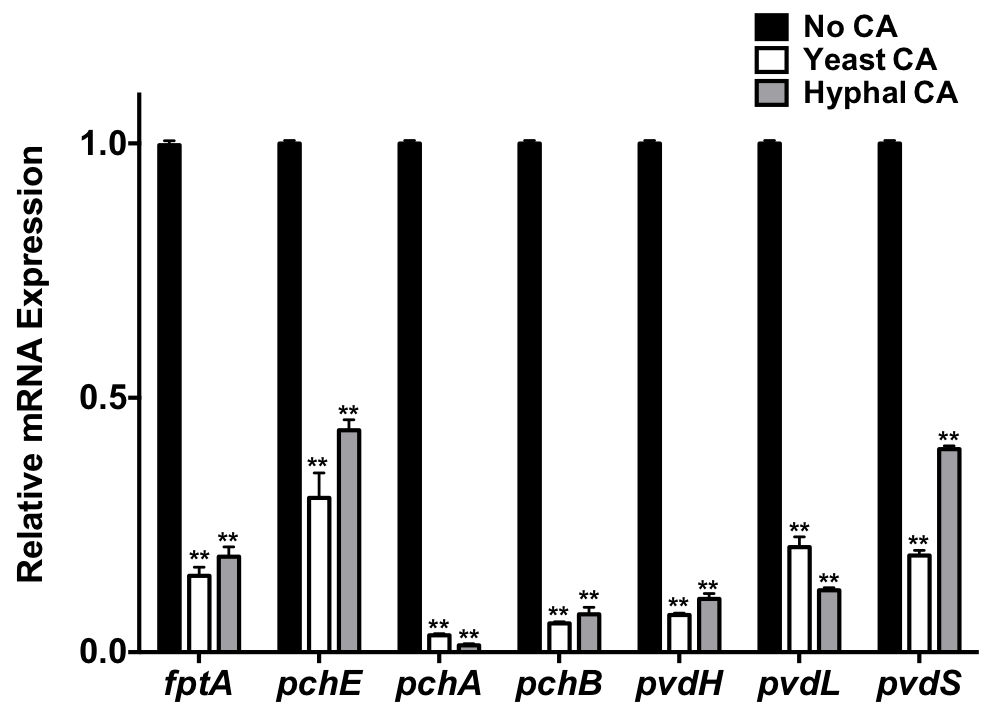

Supplement: S7 Fig — Pyochelin and pyoverdine gene expression by RT qPCR of P. aeruginosa PAO1 grown in vitro in GGP media to mid-log phase ± C. albicans (yeast), C. albicans (hyphal). Yeast C. albicans grown in YPD at 30°C. Hyphal C. albicans grown in YPD/10% fetal calf serum (FCS) at 37°C. C. albicans added to P. aeruginosa culture in 1:1 ratio and co-incubated at 37°C for 10 minutes before RNA extraction. Bars are means ± SEM. Assays were performed in triplicate. Statistical analysis was performed by unpaired Student’s t-test. * p< 0.05; ** p<0.01; ns, not significant. (TIF) [file ppat.1005129.s007.tif]

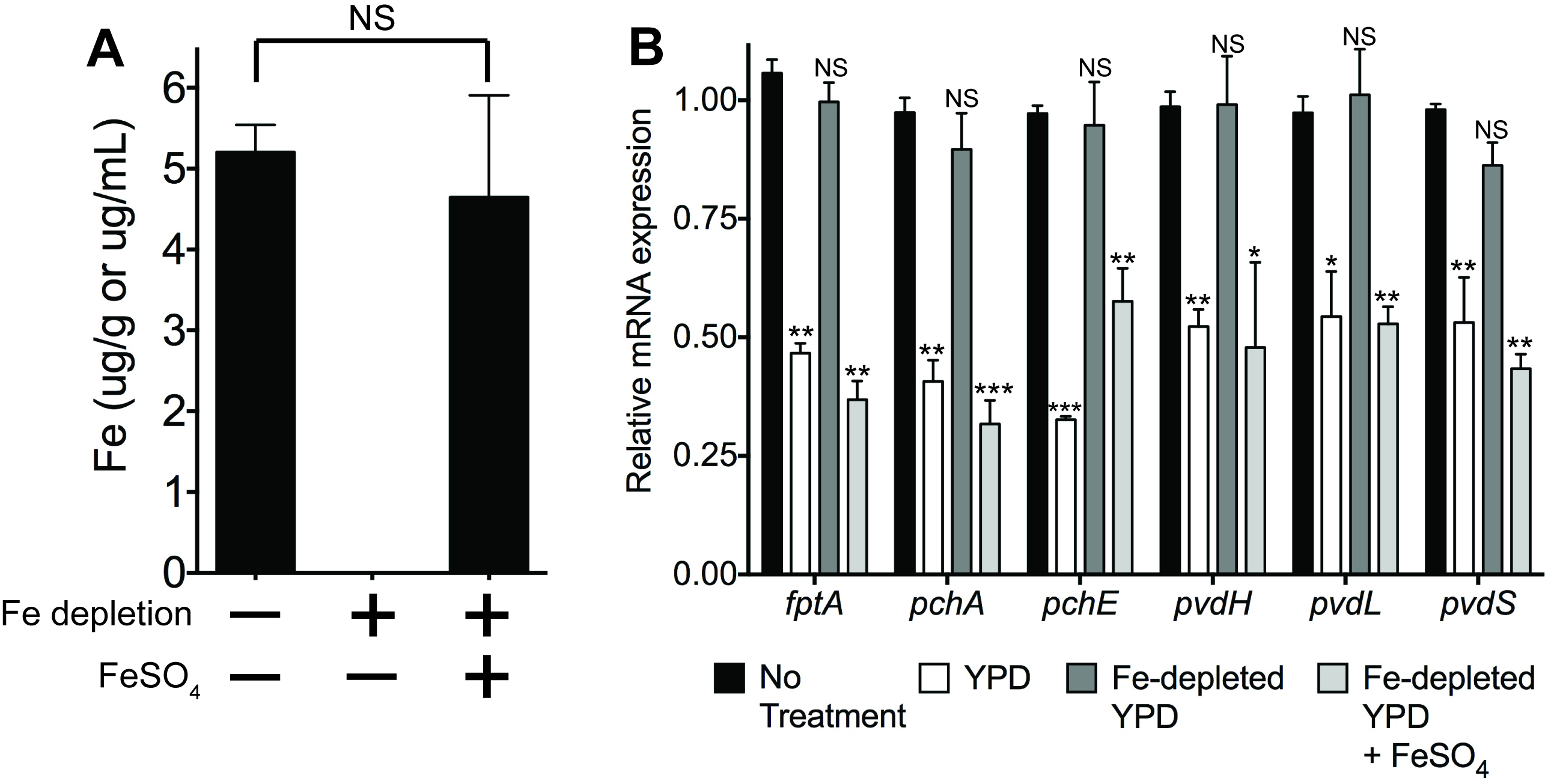

Supplement: S8 Fig — A) Total iron content of YPD media, YPD media depleted of iron (Chelex100, Sigma), and iron-depleted YPD media supplemented with iron. Total iron content (Fe2+and Fe3+) was determined by ferrozine assay. B) Pyochelin and pyoverdine gene expression by RT qPCR of P. aeruginosa PAO1 grown in vitro to mid-log phase in iron-limited GGP media with or without YPD media, YPD media depleted of iron (Chelex100, Sigma), and iron-depleted YPD media supplemented with iron. All data shown are means + SEM. Assays were performed in triplicate. Statistical analysis was performed by unpaired Student’s t-test. * p< 0.05; ** p<0.01; ns, not significant. (TIF) [file ppat.1005129.s008.tif]

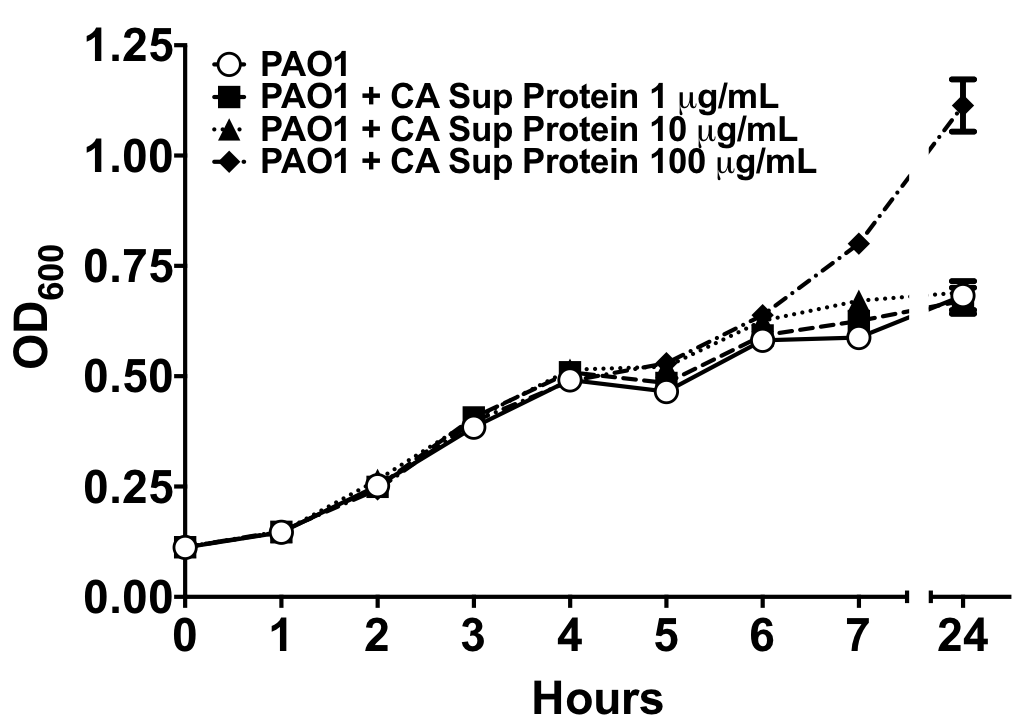

Supplement: S9 Fig — P. aeruginosa PAO1 grown in vitro in GGP media ± C. albicans supernatant proteins (0, 1, 10, and 100 μg/mL) at 37°C, 200 rpm. OD600 were measured at the time points shown. All data shown are means ± SEM. Assays were performed in triplicate. (TIF) [file ppat.1005129.s009.tif]

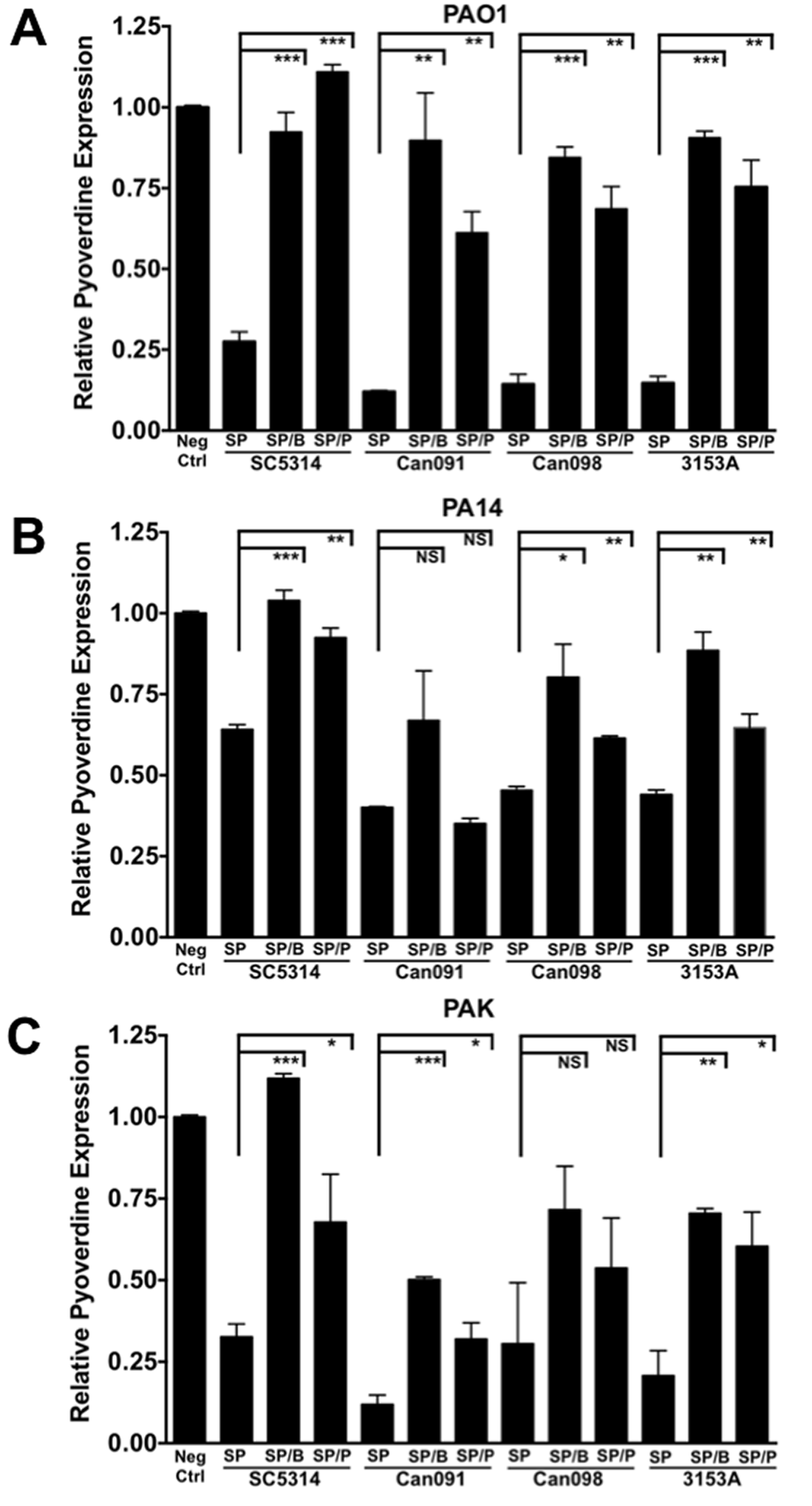

Supplement: S10 Fig — Relative pyoverdine production (as determined by measuring fluorescence at 400±10/460±40 nm excitation/emission and normalizing to cell density measured at 600 nm) of P. aeruginosa grown in GGP media at 37°C over 24 hours with C. albicans supernatant protein (SP, final concentration 100 ug/mL), boiled C. albicans supernatant protein (SP/B, boiled for 60 minutes), or C. albicans supernatant protein treated with Streptomyces griseus protease (SP/P, for 60 minutes) compared to an untreated P. aeruginosa control. A) P. aeruginosa PAO1, B) P. aeruginosa PA14, and C) P. aeruginosa PAK ± C. albicans supernatant proteins from C. albicans strains Can091, Can098, or 3153A. All data shown are means ± SEM. Assays were performed in triplicate. Statistical analysis was performed by unpaired Student’s t-test. * p< 0.05; ** p<0.01; ns, not significant. (TIF) [file ppat.1005129.s010.tif]
