# Supplementary material for: Candida albicans Inhibits Pseudomonas aeruginosa Virulence through Suppression of Pyochelin and Pyoverdine Biosynthesis
Source: PLoS Pathog. 2015 Aug 27;11(8):e1005129. doi: 10.1371/journal.ppat.1005129 (PMC4552174; doi:10.1371/journal.ppat.1005129)
Supplement: S1 Table — (PDF) [file ppat.1005129.s011.pdf]

S1 Table. Bacterial and Fungal strains used in this study

Strain	Experiment
***Candida albicans* SC5314**	CA/PA GI colonization and dissemination in antibiotic treated and germ-free mice (Fig. 1)<br>CA/*E. coli* GI colonization and dissemination (S4 Fig.)<br>*In Vivo* RNASeq Experiments (Fig. 2)<br>Iron studies (Fig. 4A)<br>*In Vitro* pyochelin and pyoverdine gene expression, pyoverdine production, and cultured colonocyte cytotoxicity experiments (Figs. 5, 6)<br>HK CA and CA secreted protein *in vivo* experiments (Fig. 6)<br>Iron-add back experiments (Fig. 7)
***Candida albicans* Can091**<br>Clinical biofilm isolate (Boston, MA, 9/00). Speciated by CHROMAgar. M. Lyon. (Provided by R. Wheeler)	CA/PA GI colonization and dissemination in antibiotic treated mice (S2 Fig., S3 Fig.)<br>Iron studies (Fig. 4A)<br>*In Vitro* pyoverdine experiments (S10 Fig.)
***Candida albicans* Can098**<br>Clinical bloodstream isolate (Hartford, CT, 3/99). Speciated by CHROMAgar, morphology and PCR. M. Lyon. (Provided by M. Lorenz)	CA/PA GI colonization and dissemination in antibiotic treated mice (S2 Fig., S3 Fig.)<br>Iron studies (Fig. 4A)<br>*In Vitro* pyoverdine experiments (S10 Fig.)
***Candida albicans* 3153A**<br>Clinical biofilm isolate [1] (Provided by M. Lorenz)	CA/PA GI colonization and dissemination in antibiotic treated mice (S2 Fig., S3 Fig.))<br>Iron studies (Fig. 4A)<br>*In Vitro* pyoverdine experiments (S10 Fig.)
P. aeruginosa PAO1	CA/PA GI colonization and dissemination in antibiotic treated and germ-free mice (Fig. 1, S1 Fig. S2 Fig., S3 Fig.)<br>PA/CA-farnesol mutants (S5 Fig.)<br>*In Vivo* RNASeq Experiments (Fig. 2)<br>PA pyochelin and pyoverdine mutants (Fig. 3, S6 Fig.)<br>*In Vitro* pyochelin and pyoverdine gene expression, pyoverdine production, and cultured colonocyte cytotoxicity experiments (Figs. 5, 6)<br>HK CA and CA secreted protein *in vivo* experiments (Fig. 6)<br>Iron-add back experiments (Fig. 7)
P. aeruginosa PAK	CA/PA GI colonization and dissemination in antibiotic treated mice (S2 Fig., S3 Fig.)<br>*In Vitro* pyoverdine experiments (S10 Fig.)

P. aeruginosa PA14	CA/PA GI colonization and dissemination in antibiotic treated mice (S2 Fig., S3 Fig.)<br>*In Vitro* pyoverdine experiments (S10 Fig.)
Bacteroidetes thetaiotamicron VPI-5482	PA/Commensal Bacteria Interactions (S1 Fig.)
Blautia producta ATCC 27340	PA/Commensal Bacteria Interactions (S1 Fig.)
Escherichia coli ATCC 10798	PA/Commensal Bacteria Interactions (S1 Fig.)<br>CA/*E. coli* GI colonization and dissemination (S4 Fig.)<br>PA gyrB qPCR specificity (Fig. 4B)<br>*In Vitro* cultured colonocyte cytotoxicity experiments (Fig. 6)
Escherichia coli, clinical isolate (bloodstream infection) (This study)	CA/*E. coli* GI colonization and dissemination (S4 Fig.)
Enterococcus faecalis, clinical isolate (bloodstream infection) (This study)	PA/Commensal Bacteria Interactions (S1 Fig.)<br>PA gyrB qPCR specificity (Fig. 4B)
Enterobacter clocae, clinical isolate (bloodstream infection) (This study)	PA gyrB qPCR specificity (Fig. 4B)

1. Murciano C, Villamon E, O'Connor JE, Gozalbo D, Gil ML (2006) Killed Candida albicans yeasts and hyphae inhibit gamma interferon release by murine natural killer cells. Infect Immun 74: 1403-1406.
