# Supplementary material for: Candida albicans Inhibits Pseudomonas aeruginosa Virulence through Suppression of Pyochelin and Pyoverdine Biosynthesis
Source: PLoS Pathog. 2015 Aug 27;11(8):e1005129. doi: 10.1371/journal.ppat.1005129 (PMC4552174; doi:10.1371/journal.ppat.1005129)
Supplement: S2 Table — (PDF) [file ppat.1005129.s012.pdf]

S2 Table. RNA-Seq Mapping Statistics

		Mapping Statistics							Paired Reads					
	# Sequences	Counted Reads	Unique	% Unique (Counted)	Non-specific	% non-specific	Uncounted Reads	Total Reads	Reads mapped in Pairs	%reads mapped in pairs	Reads mapped in broken pairs	% reads mapped in broken pairs	Reads Not mapped	Total
P1	52500961	3208752	235778	7.3479658	2972974	92.6520342	49292209	52500961	6417504	6.1117967	32284864	30.7469267	66299554	105001922
P2	56870365	2761964	199157	7.2107022	2562807	92.7892978	54108401	56870365	5523928	4.85659623	43588331	38.3225349	64628471	113740730
PC1	56988707	3335667	256695	7.6954624	3078972	92.3045376	53653040	56988707	6671334	5.85320702	35782792	31.394634	71523288	113977414
PC2	59399220	3797388	304017	8.0059504	3493371	91.9940496	55601832	59399220	7594776	6.39299304	39368613	33.1389983	71835051	118798440

P= *Pseudomonas aeruginosa*

PC = *Pseudomonas aeruginosa* and *Candida albicans*
