# Supplementary material for: Candida albicans Inhibits Pseudomonas aeruginosa Virulence through Suppression of Pyochelin and Pyoverdine Biosynthesis
Source: PLoS Pathog. 2015 Aug 27;11(8):e1005129. doi: 10.1371/journal.ppat.1005129 (PMC4552174; doi:10.1371/journal.ppat.1005129)
Supplement: S3 Table — (PDF) [file ppat.1005129.s013.pdf]

S3 Table. Primers used in this study

**Gene**	**Forward Primer**	**Reverse Primer**	**Probe**
***P. aeruginosa* pyochelin and pyoverdine qPCR**			
rpoD (PA0576)	GGGCGAAGAAGGAAATGGTC	CAGGTGGCGTAGGTGGAGAA	
fptA (PA4221)	CGGCGTGCTGGTGTAGGTGTAGCCG	CGCCCAACAATCCGTTCTACAT	
pchA (PA4231)	CTGCCTGTACTGGGAACAGC	GCAGAGCAATTGCCAGTTTT	
pchB (PA4230)	CGAGGAAAACGGACTCGAC	GCGCCAGTACTTGATCTGCT	
pchE (PA4226)	CGGAATTTCTCCTCAACGTG	AGCAACAGGGTGGTGAAGTC	
pchF (PA4225)	CGATTAGGCTCGCTCTCCAGGTAATCG	CCATCACCCTGTGGAACTCG	
pchG (PA4224)	CTGATCGAGCATCCGTTG	GCGGATAGAAGGTGTTGAGC	
pchH (PA4223)	ACTGAAGGACATGCCTGATG	GAACAGGGCAATGTTGTCG	
pvdS (PA2426)	ACCGTACGATCCTGGTGAAG	TGAACGACGAAGTGATCTGC	
pvdL (PA2424)	CCAGCTCGACTACTGGAAGG	CTCAGGTCGAAGCGGTAGAG	
pvdH (PA2413)	CAGTTCCTGCCGTATCCCTA	GGATCGTTCAGCAGGTTCTC	
pchD (PA4228)	GCGACAAGGACCAGATCAAC	GATGGGCTATCAGCAGGTTC	
ampP (PA4218)	CTGGGTCGACAATCACTGGT	GATCTGTACCGCGTTGACCT	
***P. aeruginosa gyrB* qPCR**			
gyrB (PA0004)	CAGGAGCAGTACATCAAGGAC	TTGACCAGTTTCTCCAGCG	ATGGAAGAGTACATGACCCAG TCGG
