# Supplementary material for: Candida albicans Inhibits Pseudomonas aeruginosa Virulence through Suppression of Pyochelin and Pyoverdine Biosynthesis
Source: PLoS Pathog. 2015 Aug 27;11(8):e1005129. doi: 10.1371/journal.ppat.1005129 (PMC4552174; doi:10.1371/journal.ppat.1005129)
Supplement: S4 Table — (PDF) [file ppat.1005129.s014.pdf]

S4 Table. ***Pseudomonas aeruginosa*** **strains and plasmids used in this study to generate PA pyochelin/pyoverdine knockout mutants**

Name	Relevant Characteristics	Reference
E. coli **strains**		
DH5 α	*recA1 endA1 hsdR17 deoR thi-1 supE44 gyrA96 relA1 Δ(lacZYA-argF) U169 (Φ80dlacZΔM15)*	Sambrook & Russell (2001)
P. aeruginosa **strains**		
PAO1	Wild-type	ATCC 15692
PAO6297	PAO1 Δ *pchBA*	Reimmann et al. (1998)
PAO6310	PAO1 Δ *pchE*	Reimmann et al. (1998)
PAO6428	PAO1 Δ *fptA*	Michel et al. (2007)
PAO6382	PAO1 Δ *pvdF*	Hoegy et al. (2009)
PAO6383	PAO1 Δ *pchBA* Δ *pvdF*	Michel et al. (2007)
PAO6901	PAO1 *pvdS*::Gm^r	This study
PAO6902	PAO1 *pvdS*::Gm^r Δ *pchBA*	This study
PA258	PAO1 Δ *pvdH*	This study
PA259	PAO1 Δ *pchBA* Δ *pvdH*	This study
**Plasmids**		
pME6000	pBBR1 ori, Tet^r *lacZ mob*$^+$	Maurhofer et al. (1998)
pME6477	pME6000 derivative carrying *pchDCBA*, Tet^r	This study

Tet^r, tetracycline resistance; Gm^r, gentamicin resistance

Hoegy, F., Lee, X., Noel, S., Rognan, D., Mislin, G. L., Reimmann, C. & Schalk, I. J. (2009). Stereospecificity of the siderophore pyochelin outer membrane transporters in fluorescent pseudomonads. *J Biol Chem* 284, 14949-14957.

Maurhofer, M., Reimmann, C., Schmidli-Sacherer, P., Heeb, S., Haas, D. & Défago, G. (1998). Salicylic acid biosynthesic genes expressed constitutively in the root-colonizing *Pseudomonas fluorescens* strain P3 improve the induction of systemic resistance in tobacco against tobacco necrosis virus. *Phytopathology* 88, 678-684.

Michel, L., Bachelard, A. & Reimmann, C. (2007). Ferripyochelin uptake genes are involved in pyochelin-mediated signalling in *Pseudomonas aeruginosa*. *Microbiology* 153, 1508-1518.

Reimmann, C., Serino, L., Beyeler, M., & Haas, D. (1998) Dihydroaeruginoic acid synthetase and pyochelin synthetase, products of the *pchEF* genes are induced by extracellular pyochelin in *Pseudomonas aeruginosa*. *Microbiology* 144, 3135-3148.

Sambrook, J. & Russell, D. W. (2001). *Molecular cloning: a laboratory manual, 3rd edition*. Cold Spring Harbor, N.Y.: Cold Spring Harbor Laboratory.
